# Machine Learning Uncovers Natural Product Modulators of the 5-Lipoxygenase Pathway and Facilitates the Elucidation of Their Biological Mechanisms

**DOI:** 10.1021/acschembio.3c00725

**Published:** 2023-12-27

**Authors:** Sigitas Mikutis, Stefanie Lawrinowitz, Christian Kretzer, Lavinia Dunsmore, Laurynas Sketeris, Tiago Rodrigues, Oliver Werz, Gonçalo J. L. Bernardes

**Affiliations:** † Yusuf Hamied Department of Chemistry, University of Cambridge, Lensfield Road, Cambridge CB2 1EW, U.K.; ‡ Department of Pharmaceutical/Medicinal Chemistry, Institute of Pharmacy, Friedrich Schiller University Jena, Philosophenweg 14, 07743 Jena, Germany; § Instituto de Investigação do Medicamento (iMed), Faculdade de Farmácia, Universidade de Lisboa, Av. Prof. Gama Pinto, 1649-003 Lisbon, Portugal; ∥ Instituto de Medicina Molecular João Lobo Antunes, Faculdade de Medicina, Universidade de Lisboa, Avenida Professor Egas Moniz, 1649-028 Lisboa, Portugal

## Abstract

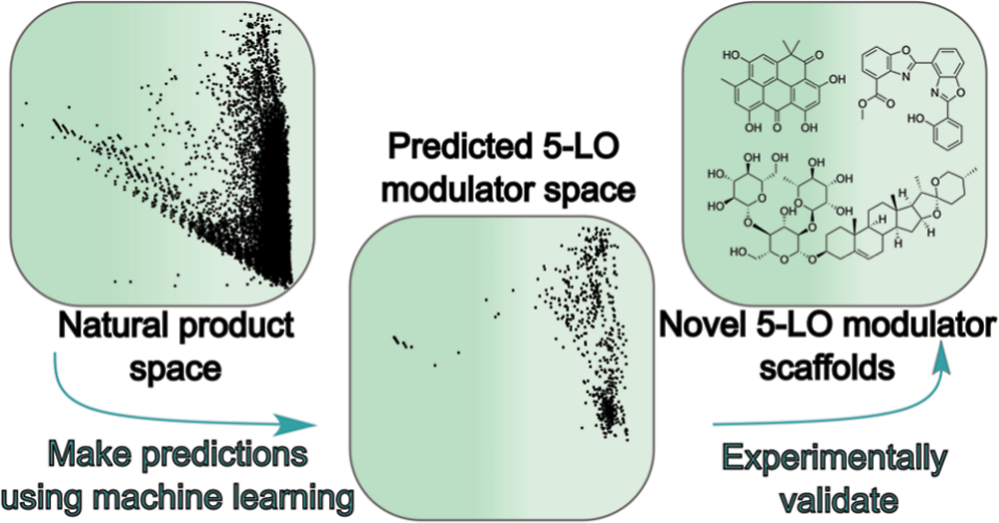

Machine learning (ML) models have made inroads into chemical sciences, with optimization of chemical reactions and prediction of biologically active molecules being prime examples thereof. These models excel where physical experiments are expensive or time-consuming, for example, due to large scales or the need for materials that are difficult to obtain. Studies of natural products suffer from these issues—this class of small molecules is known for its wealth of structural diversity and wide-ranging biological activities, but their investigation is hindered by poor synthetic accessibility and lack of scalability. To facilitate the evaluation of these molecules, we designed ML models that predict which natural products can interact with a particular target or a relevant pathway. Here, we focused on discovering natural products that are capable of modulating the 5-lipoxygenase (5-LO) pathway that plays key roles in lipid signaling and inflammation. These computational approaches led to the identification of nine natural products that either directly inhibit the activity of the 5-LO enzyme or affect the cellular 5-LO pathway. Further investigation of one of these molecules, deltonin, led us to discover a new cell-type-selective mechanism of action. Our ML approach helped deorphanize natural products as well as shed light on their mechanisms and can be broadly applied to other use cases in chemical biology.

## Introduction

The past decade has seen a rise in use of machine learning (ML) models for molecular applications. Some of their applications include prediction of reaction yields,^1^ optimization of molecular dynamics calculations,^2^ and inference of small-molecule bioactivity.^3^ The latter is a particularly challenging task as it needs to predict complex interactions between large biomolecules and their modulators. To highlight some examples, Zhavoronkov and colleagues used generative ML models that helped to design de novo inhibitors of DDR1 kinase,^4^ Stokes and colleagues reported a deep learning-based platform to screen for antibiotics in large data sets of molecules,^5^ whereas Svensson and colleagues utilized random forests (RFs) and conformal predictions to assess the cytotoxicity of chemical matter against 16 cell lines.^6^ These examples highlight the potential of ML to streamline chemical biology and minimize experimentation.

One field of research where ML models could make a difference is the deorphanization of natural products—molecules that often exhibit complex biological activities and possess a wide variety of chemical motifs. Indeed, a number of studies report ML models able to predict the properties of natural products including antimicrobial, anticancer, and anti-inflammatory activities as well as abilities to modulate protein expression.^7,8^ Natural products are well-known for their polypharmacology,^9^ i.e., they can often interact with multiple biomolecules, which leads to complex bioactivities. The chemical space occupied by natural products is different from that of molecules produced in medicinal chemistry campaigns, and functionalizing clinical candidates with natural products motifs can lead to more potent biomodulators.^10^ Thus, the study of natural products can lead to the discovery of new therapeutically relevant chemical matter, uncover new mechanisms of action, and, in many cases, directly lead to new pharmaceuticals. Indeed, 396 natural products or their derivatives (mostly semisynthetically modified natural products) were approved by the FDA in 1981–2014, so they remain an important class of new therapeutics.^11^ However, the chemical space covered by natural products is wide, and for many, it is difficult or even impossible to obtain amounts sufficient for comprehensive testing. As such, in silico approaches to predict the properties of natural products are highly desirable and can help to streamline the use of scarce resources.

For this aim, we went on to develop ML models to discover natural product modulators of a particular biochemical process, which as a result could be suitable as a chemical tool. Our approach utilizes publicly available small molecule–biomolecule interaction databases ChEMBL,^12^ data from which are used as the basis for predictive models, and PubChem,^13^ used to further refine the list of predicted modulators. We used the ML models to predict the modulators of the 5-lipoxygenase (5-LO) pathway, which plays a role in the inflammatory response.^14−16^ 5-LO uses a nonheme iron to oxidize essential fatty acids, in particular arachidonic acid (AA), with leukotrienes (LT) being one of the key products; thus, 5-LO plays a central role in lipid signaling. A number of other proteins contribute to cellular 5-LO product formation, forming a complex network of interactions. These proteins include the cytosolic phospholipase (cPL)A_2_ that releases AA, 5-LO-activating protein (FLAP) that facilitates the access of 5-LO to AA, as well as LTA_4_ hydrolase and LTC_4_ synthase that generate the downstream LTB_4_ and LTC_4_, respectively. Moreover, the activity of the 5-LO pathway is prone to complex regulation by Ca^2+^, peroxides, phospholipids, and phosphorylation events. 5-LO-derived products are of relevance for various inflammatory and allergic disorders, with one small-molecule 5-LO inhibitor—zileuton—being approved against asthma.^17^ 5-LO also plays an important role in acute myeloid leukemia, as recently disclosed by us.^18,19^ Thus, it is conceivable that some natural products with anti-inflammatory properties could modulate the activity of this pathway. We were especially interested in discovering natural products that would constitute a novel 5-LO modulator chemical space. To accomplish this, we elected to use physiochemical descriptors to build the ML models rather than molecular fingerprints or other structure-based methods as they would make predictions solely on the basis of the structural features.

The ML models we build led us to test 21 natural products against 5-LO. Due to the nature of the training data, we were able to discover new direct enzyme and cellular pathway inhibitors of 5-LO, highlighting the polypharmacology expressed by these molecules. A number of these natural products indeed modulated 5-LO product formation, which highlights the suitability of our approach to discovering natural products modulating a particular pathway. We carried out a more in-depth characterization of deltonin and discovered that it modulates the 5-LO pathway in a cell type-dependent manner, with a mechanism that involves elevation of the intracellular Ca^2+^ level and suppression of ROS modulation in human polymorphonuclear leukocytes (PMNLs). We believe that further study of this mechanism can provide new therapeutic insights, especially regarding cell type selectivity.

## Methods

### Data Preparation

5128 entries describing the small-molecule modulation of 5-LO and 4781 entries describing the modulation of FLAP were downloaded from the CHEMBL23 database and uploaded into the KNIME analytics platform (v. 3.4.0).^20^ Only entries that report exact IC_50_, *K*_D_, or *K*_i_ relationships at nanomolar concentrations were kept, and duplicates were removed, leading to 2456 and 2245 entries for 5-LO and FLAP data sets. Molecules with reported activities below 1000 nM were classified as strong modulators (label: 1), with the rest labeled as weak (label: 0). RDKit descriptors (117) were calculated for each molecule and were then normalized.

### ML Models

No-code ML models were built with native nodes in the KNIME 3.4.0 environment using the normalized descriptors of 2456 5-LO and 2245 FLAP modulators. A 10-fold cross-validation was performed to optimize hyperparameters and gauge the performance of three classifiers: gradient-boosted trees (GBT),^21^ logistic regression (LR),^22^ and naïve Bayesian (NB)^23^ models according to the true negative rate (TNR), positive predictive value (PPV), the Matthew’s correlation coefficient, and balanced accuracy (BA),^24^ defined as
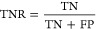
1
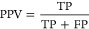
2
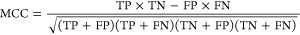
3
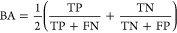
4where TP—number of true positives, TN—number of true negatives, FP—number of false positives, and FN—number of false negatives. We use 1234 as the random state. The relevant hyperparameters for the 5-LO models are as follows: GBT—tree depth = 4, number of trees = 500, learning rate = 0.05; LR—maximal number of epochs = 100, epsilon = 1.0 × 10^–5^, step size = 0.1; NB—default probability = 0.0, and maximum number of unique nominal values per attribute = 20. For FLAP models, the parameters are the same except GBT—learning rate = 0.15, and NB—default probability = 0.005.

### Predicting 5-LO Natural Product Inhibitors

A data set of 24,500 commercially available natural products (an overlap between DNP and zinc commercially available databases) was uploaded into the KNIME analytics platform. Molecules were preprocessed, and the descriptors were calculated and normalized as in the training set. The implemented GBT, LR, and NB models were used to predict labels for the test set, leading to 1245 molecules predicted as positives by all three models and further 2254 predicted by any two models (full metrics in Table S1) for the 5-LO data set. For FLAP data, only the 940 triple-positive predictions were considered. Triple-positive and part of random (500 out of 2254 double-positive 5-LO set molecules) were further investigated in the PubChem database for their anti-inflammatory, antileukemia, and antiarthritic activities. Eleven and ten randomly chosen natural products from 5-LO and FLAP data sets, respectively, with known anti-inflammatory, antileukemic, or antiarthritic properties were selected for further testing. Molecules described as 5-LO modulators in the ChEMBL database were not chosen for testing.

### Fragment Analysis

2456 5-LO modulators were split into strong and weak modulator data sets as described above. Molecules in both sets were broken down into fragments with path lengths 4–8. Fragments containing interrupted aromatic systems were discarded. Fragment frequencies (number of molecules containing a particular fragment in a set vs total number of molecules in a set) in both sets were calculated. Ratio frequency(active)/Frequency(inactive) was used to calculate the relative representation of each fragment in the active and inactive data sets.

### Similarity Search

The natural products chosen for testing and all the 5-LO modulators in the ChEMBL23 database were converted into RDKit (2048 bits, min path length = 1, max path length = 7) and AtomPair^25^ (2048 bits, min path length = 1, max path length = 30) molecular fingerprints. Tanimoto similarity search was carried out between the fingerprints of chosen natural products and known 5-LO modulators in order to find the most similar molecules between the two sets.

### p*K*_a_ Predictions

p*K*_a_ values of resistomycin were predicted using MarvinView (ChemAxon/Infocom Marvin Extensions Feature 4.4.0.v211500) in the KNIME Analytics Platform.

### Expression and Purification of Human Recombinant 5-LO

Human recombinant 5-LO was expressed in *Escherichia coli* (BL21, DE3) transformed with the pT3-5-LO plasmid at 30 °C overnight, as described before.^26^ Cells were lysed using lysis buffer and homogenized by sonification (3 × 15 s, Branson Sonifier 250, Branson Ultrasonics Corporation). 5-LO was then purified from 40,000*g* supernatant (20 min, 4 °C) using an ATP agarose column (Sigma-Aldrich), diluted with PBS (Dulbecco’s formula, pH 7.4) buffer containing 1 mM EDTA, and immediately used for 5-LO activity assays.

### Blood Cell Isolation

The process of isolation was performed as described by A. Boyum.^27^ Briefly, peripheral blood was withdrawn from fasted healthy adult volunteers (University Hospital Jena, Germany) and centrifuged to obtain leukocyte concentrates. These were aliquoted and mixed with 2.5% dextran in PBS. After 45 min, the leukocyte-rich supernatant was transferred to a density centrifugation medium (Histopaque-1077; *d* = 1.077) and centrifuged (200 rpm, 10 min, RT, without brake, Heraeus Multifuge X3R Centrifuge, Thermo Fisher Scientific).

Peripheral blood mononuclear cells (PBMC) were concentrated on top of the density medium and separated from the other cells. Isolated PBMC were washed with PBS twice (1200 rpm, 5 min, 4 °C) and resuspended in 5 mL of PBS.

For the isolation of PMNLs, contaminating erythrocytes of pelleted PMNLs were removed by hypotonic lysis. Afterward, PMNLs were washed with PBS twice (1200 rpm, 5 min, 4 °C) and resuspended in 5 mL of PBS. A cell counting system (Vi-CellTMXR, Beckmann Coulter) was used to determine the cell numbers and cell viability. For counting, the cell suspension was diluted (1:50) and trypan blue staining [0.4% (v/v), sterile filtered] was used to determine cell viability.

### Determination of 5-LO Product Formation Using Isolated Enzyme

Isolated human 5-LO was diluted to optimal concentration (approximately 0.5 μg/mL) in 1 mL of PBS containing 1 mM EDTA and treated with compounds or vehicle [0.1% (v/v) DMSO] for 10 min at 4 °C, and stimulated by the addition of 2 mM CaCl_2_ and 20 μM AA for 10 min at 37 °C. The reaction was stopped by adding 1 mL of ice-cold methanol, and samples were prepared for high-performance liquid chromatography (HPLC) analysis.

### Determination of 5-LO Product Formation in PMNL

Freshly isolated PMNLs (5 × 10^6^) were suspended in 1 mL of PBS-glucose (Dulbecco’s PBS with 1% w/v glucose) buffer, and 1 mM CaCl_2_ was added. Cells were treated with compounds or vehicle [0.1% (v/v) DMSO] for 10 min at 37 °C and stimulated by the addition of 2.5 μM A23187 (and 20 μM exogenous AA as stated in the text) for 10 min at 37 °C. The reaction was stopped by adding 1 mL of ice-cold methanol, and samples were prepared for HPLC analysis.

### Solid-Phase Extraction and HPLC Analysis

After stopping the 5-LO reactions (see above), 530 μL of PBS-HCl (500 μL of Dulbecco’s PBS mixed with 30 μL of 1 N HCl aqueous solution) and 200 ng of internal standard PGB_1_ were added to the samples. The samples from PMNL incubation were centrifuged (870*g*, 10 min, 4 °C) before solid-phase extraction (SPE) was performed using reversed-phase C18 SPE cartridge clean-up (United Chemical Technologies). Briefly, columns were conditioned with methanol and ddH_2_O, samples were added, washed with ddH_2_O and 25% (v/v) methanol, eluted with 300 μL of methanol, mixed with 120 μL of ddH_2_O; 100 μL were then subjected to analysis. Major 5-LO products [LTB_4_, all-trans isomers of LTB_4_, and 5-H(p)ETE] were analyzed by RP-HPLC using a Nova-Pak C18 Radial-Pak Column (4 μm, 5 × 100 mm, Waters) under isocratic conditions (73% methanol/27% water/0.007% trifluoroacetic acid) at a flow rate of 1.2 mL/min and detected at 235 nm [for 5-H(p)ETE] or 280 nm (for LTB_4_ and its all-trans isomers).

### Lactate Dehydrogenase Release Assay

The lactate dehydrogenase (LDH) assay was performed using the CytoTox 96 Nonradioactive Cytotoxicity Assay kit. Briefly, 2 × 10^5^ PMNL or PBMC diluted in PBS-glucose buffer were seeded per well of a 96-well plate. Lysis control and 0.2% (v/v) triton X-100 were added to the cells and incubated for 45 min; compounds and vehicle [0.1% (v/v) DMSO] were added and incubated for 20 min at 37 °C. A stop solution was added, the plate was centrifuged (250*g*, 4 min, RT), and 50 μL of supernatant from each well was transferred. Afterward, 50 μL of the substrate mixture was added and incubated for 30 min at RT under the exclusion of light. To finally stop the reaction, 50 μL of the stop solution was added, and the photometric measurement was done at 490 nm using a Multiscan Spectrum plate reader (Thermo Fisher Scientific). Cytotoxicity was calculated after background correction as
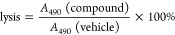
5

### Hemolysis Assay

Defibrinated Oxoid sheep’s blood (Thermo Fisher Scientific, Waltham, MA, USA) was diluted to a 5% (v/v) suspension in PBS. In a 96-well microtiter plate, 190 μL of the blood suspension was added to 10 μL of compound in PBS containing 20 × stock. DMSO was diluted in PBS to a 20 × stock, which after adding the blood gave a concentration of 0.1% (v/v, negative control). Addition of 1% (v/v) triton X-100 was used as a positive hemolysis control. Three replicates were performed for each compound concentration. The plate was then incubated at 37 °C for 2 or 24 h. Following the incubation, the plate was centrifuged (3300 rpm, 5 min), and 100 μL of the supernatant was collected and transferred. To determine hemolysis, ultraviolet (UV) absorbance of free heme was measured immediately at 540 and 500–700 nm (spectrum) using a plate reader. Percentage of hemolysis was determined by
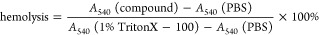
6

### MTT Assay

Freshly isolated PBMC were diluted in RPMI supplemented with 10% fetal calf serum, 1% penicillin/streptomycin, and 1 mM l-glutamine, and 100 μL of the resulting cell suspension containing 2 × 10^5^ cells was seeded per well in a 96-well plate. A 333-fold stock of compound or 0.3% (v/v) DMSO was added and incubated for 1 or 24 h at 37 °C. As a positive control, 16.7% (v/v) ethanol or 0.05% (v/v) triton-X was used; a negative control contained media only. Finally, 20 μL of MTT solution (5 mg mL^–1^ in PBS, sterile-filtered) was added per well and incubated for 3 h at 37 °C. Cells were lysed by the addition of 100 μL of SDS lysis buffer with shaking at 175 rpm (neoLab Multi shaker DOS-102, neoLab Migge) under the exclusion of light overnight. The photometric measurement was performed at 400–600 nm (595 nm) using a Multiscan Spectrum plate reader (Thermo Fisher Scientific). Cell viability was calculated after background correction as
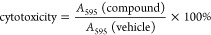
7

### Cultivation and Preparation of Human Pathogenic *E. coli*

Human pathogenic *E. coli* (O6:K2:H1 CFT073) was cultivated in 35 mL of NB medium suspension in an Erlenmeyer flask with shaking at 37 °C overnight. On the next day, 5 mL of cell suspension was centrifuged (5000*g*, 5 min, 20 °C), and the optical density OD600 was brought to 1.0 by diluting cells in PBS (pH 7.4) supplemented with 1 mM CaCl_2_. OD_600_ was determined using an Ultrospec10-Cell density meter (Amersham Biosciences).

### Differentiation and Polarization of Monocytes to M1 Macrophages

Freshly isolated PBMC were cultivated in 15 mL of PBS (Dulbecco’s formula, supplemented with 100 mg/L CaCl_2_ and MgCl_2_ hexahydrate) in a 75 cm^3^ cell culture flask at 37 °C for 1 h in the WTC Binder incubator (WTC Binder GmbH) in a humid and CO_2_-enriched (5% v/v) atmosphere. After attachment of monocytes, cells in suspension were removed and 15 mL of RPMI supplemented with 10% fetal calf serum, 1% penicillin/streptomycin, and 1 mM l-glutamine medium were added per flask. For macrophage differentiation, 20 ng/mL of GM-CSF was supplemented, and after 6 days, cells were polarized with 20 ng/mL of interferon-γ and 100 ng/mL of LPS for 48 h at 37 °C and 5% (v/v) CO_2_.

### Stimulation of M1 Macrophages and Lipid Mediator Profiling by UPLC–MS/MS

M1 macrophages (1 × 10^6^) were resuspended in 1 mL of PBS (Dulbecco’s formula, supplemented with 1 mM CaCl_2_) and treated with test compounds or vehicle [0.1% (v/v) DMSO] for 20 min at 37 °C and 5% (v/v) CO_2_. Afterward, the macrophages were stimulated by the addition of *E. coli* (O6:K2:H1) at a ratio of 1:50 [M1:*E. coli* multiplicity of infection (MOI) of 50] for 90 min at 37 °C and 5% (v/v) CO_2_. The reaction was stopped by transferring the supernatant to 2 mL of ice-cold methanol, and samples were prepared for ultraperformance liquid chromatography–tandem mass spectrometry (UPLC–MS/MS) analysis.

For stimulation with Ca^2+^-ionophore A23187, the medium was removed, and the macrophages (1 × 10^6^) were treated by addition of compounds or vehicle [0.1% (v/v) DMSO] diluted in 1 mL of PBS (Dulbecco’s formula, supplemented with 1 mM CaCl_2_) for 10 min at 37 °C and 5% (v/v) CO_2_. Stimulation was performed by the addition of 2.5 μM A23187 for 10 min at 37 °C and 5% (v/v) CO_2_. The reaction was stopped by transferring the supernatant to 2 mL of ice-cold methanol, and samples were prepared for UPLC–MS/MS analysis.

To the samples were added deuterated lipid mediators (d8-5S-HETE, d4-LTB_4_, d5-LXA_4_, d5-RvD2, and d4-PGE_2_; 200 nM each) and d8-AA (10 μM) as internal standards. Lipid mediators were extracted by SPE using Sep-Pak C18 6 cm^3^ Vac Cartridges (500 mg; Waters), as previously described.^28^ Briefly, the samples were stored at −20 °C for at least 45 min to allow protein precipitation. After centrifugation (1200*g*, 4 °C, 10 min), the supernatant was combined with acidified water (pH 3.5, 7 mL) and loaded onto pre-equilibrated solid-phase cartridge columns. Washing steps with water and *n*-hexane (6 mL, each) followed. Lipid mediators were eluted with methyl formate (6 mL), brought to dryness using an evaporation system (TurboVap LV, Biotage, Uppsala, Sweden), and resuspended in methanol/water (50/50, v/v, 100 μL) for UPLC–MS/MS analysis.

For metabololipidomics analysis, lipid mediators were separated at 50 °C on an Acquity UPLC BEH C18 column (130 Å, 1.7 μm, 2.1 mm × 100 mm, Waters). The Acquity Ultraperformance LC system (Waters) was operated at a flow rate of 0.3 mL/min using a mobile phase consisting of methanol, water, and acetic acid (42:58:0.01, v/v/v), which was ramped to 86:14:0.01 (v/v/v) over 12.5 min followed by isocratic elution at 98:2:0.01 (v/v/v) for 3 min.^28^ Eluted lipid mediators were detected by (scheduled) multiple reaction monitoring using a QTRAP 5500 mass spectrometer (Sciex, Framingham, MA), which was equipped with an electrospray ionization source that was operated in negative mode. Acquired mass spectra were processed using Analyst 1.6.2 (Sciex).

### Determination of the Intracellular Ca^2+^ Concentration

The determination of the intracellular Ca^2+^ concentration was performed as previously described.^29^ Briefly, 5 × 10^6^ PMNL or 1 × 10^6^ M1 macrophages were stained using 1 μM Fura-2-AM (30 min, 37 °C), washed with Krebs-HEPES buffer (centrifuged at 1000 rpm, 5 min, 4 °C), and resuspended in 3 mL of ice-cold Krebs-HEPES buffer plus BSA. 200 μL aliquot of cell suspension containing 5 × 10^5^ PMNL or 2.5 × 10^5^ M1 macrophages was seeded per well into a 96-well plate (black, clear bottom). Cells were incubated with 1 mM CaCl_2_ for 10 min at 37 °C. To determine intracellular Ca^2+^ concentrations, a microplate fluorometer (NOVOstar, BMG Labtech Optima) was used to measure the Ca^2+^-dependent fluorescence of the intracellular dye over time at 37 °C. Fluorescence at 340 nm (Ca^2+^ chelating Fura-2-AM) and 380 nm (free Fura-2-AM) was measured every 1.18 s for 225 kinetic cycles (4.4 min total time). An automatic injecting system was used to perform the addition of the following solutions: 2 μL of 100-fold compound solution or vehicle [1% (v/v) DMSO] was injected after 11.8 s, 20 μL of 10% (v/v) triton-X in Krebs-HEPES buffer plus BSA was injected after 218.3 s, and 16.6 mM of EDTA was added after 253.7 s. Each treatment was performed in duplicates. Intracellular Ca^2+^ was calculated from the ratio of fluorescence at 340/380 nm. Maximal Ca^2+^ release was calculated as
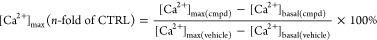
8

### Determination of Intracellular ROS Formation

The detection of ROS was conducted using peroxide-sensitive fluorescence dye DCFH-DA. PMNLs were diluted to 5 × 10^6^ cells/mL in PBS-glucose buffer and 100 μL per well were seeded into a 96-well plate (black, clear bottom). Compound, vehicle [2% (v/v) DMSO] or DPI (positive control inhibitor of ROS formation), and 100 μL of ROS measuring solution (2 μg/mL DCFH-DA, 2 mM CaCl_2_, in PBS-glucose buffer) were added and incubated for 10 min at 37 °C under the exclusion of light. Afterward, either PBS-glucose buffer or 1 μM PMA was added, and ROS formation was measured immediately (*t*0) at 37 °C using a microplate fluorometer (NOVOstar, BMG Labtech Optima). Excitation occurred at 485 nm, where emission was measured at 520 nm every 6 s for 150 kinetic cycles (15 min total time). The detection level was reached after 600 s, and thus, evaluation was performed after 480 s (*t*1) when the curve was still in the linear range. ROS formation was calculated from the fluorescence change (*t*1 – *t*0) as a percentage of vehicle control; values of cells treated with vehicle and stimulated with PMA were set as 100%. Calculation was performed as
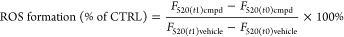
9

### Statistical Analysis

Data are presented as the mean ± SD of *n* observations, where *n* represents the number of independent experiments performed at different time points. General statistical analyses were carried out using a two-sided Student’s *t*-test at a confidence interval of 95%.

## Results and Discussion

### Building ML Models for the Discovery of 5-LO Pathway Modulators

**Figure 1:**
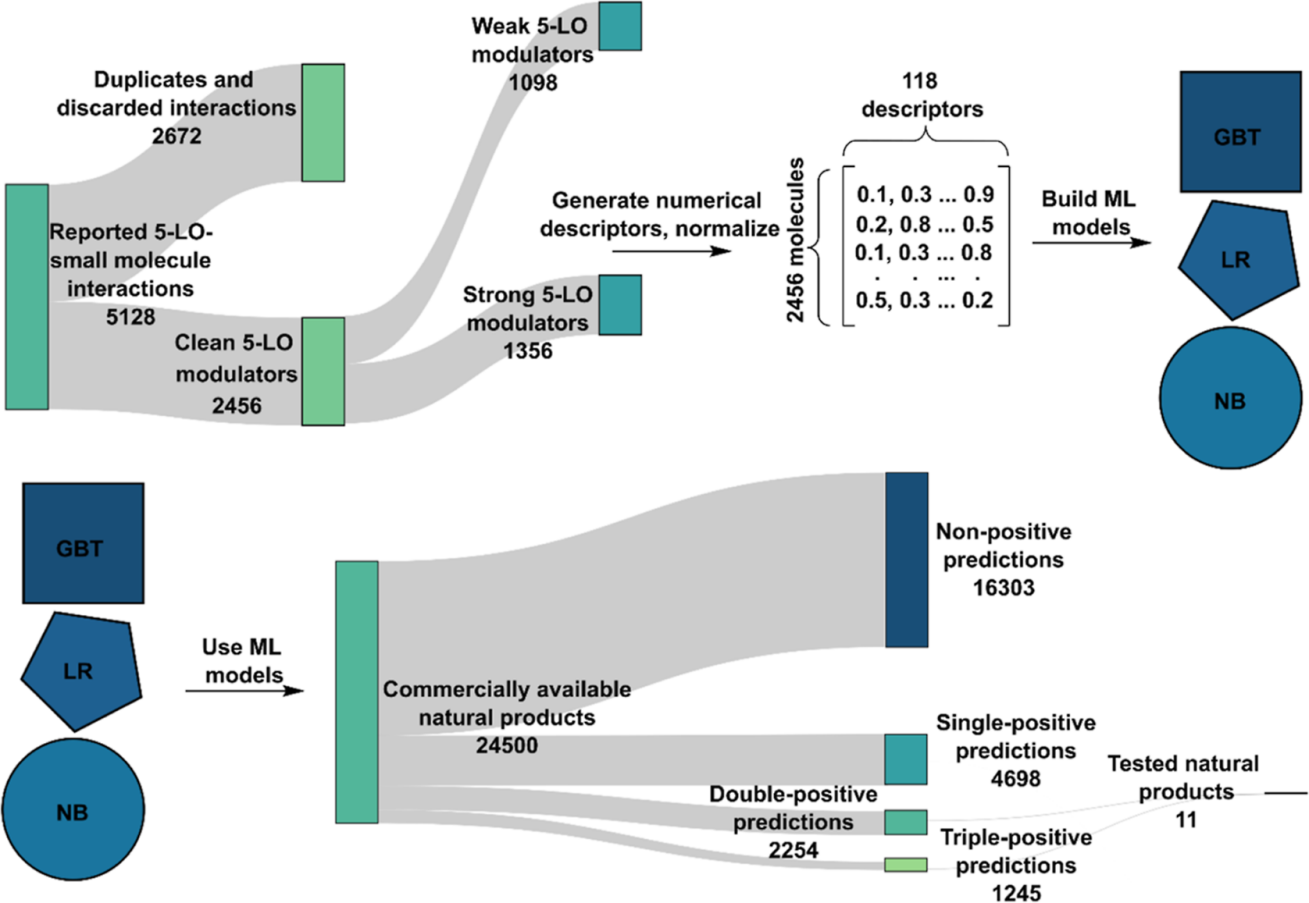
Workflow of predicting natural product 5-LO inhibitors. Known small-molecule—5-LO interactions were cleaned up; the molecules were stratified into strong and weak/nonmodulators (cutoff IC_50_ = 1 μM). Numerical structure descriptors were generated for each molecule. Three predictive classification models were built (gradient boosted trees, logistic regression, and naive Bayesian) based on the descriptors. The models were then used to predict the activities of 24,500 commercially available natural products and natural product derivatives. A subset of triple- and double-positive predictions were further investigated in the PubChem database. Eleven natural products with bioactivities that could be consistent with 5-LO inhibition were chosen for further testing. A similar approach was taken to predict 5-LO pathway inhibitors from the data set of FLAP activity modulators, as described in the text.

Suppression of cellular 5-LO product [LTA_4_, 5-H(P)ETE] formation can be achieved by the inhibition of cPLA_2_, FLAP, and 5-LO and by the modulation of cofactors such as Ca^2+^, ROS/hydroperoxides, MAPK kinases, and phospholipids/glycerides^30^ that are collectively referred here to as the “5-LO pathway”. With the goal of developing a tool that would help identify modulators of the 5-LO pathway, we collected and curated ChEMBL 23 data pertaining to the targets of interest. First, we collected data on 5-LO inhibitors. 5128 entries describing small molecules inhibiting the 5-LO enzyme were downloaded from the CHEMBL 23 database, which were then cleaned by removing duplicates and only keeping the relationships that declared exact IC_50_, *K*_D_, or *K*_i_ values, trimming down the set to 2456 5-LO inhibitors (Figure 1). For building the models, compounds with IC_50_/*K*_D_/*K*_i_ values below 1 μM were classified as strong inhibitors, and the rest were defined as weak/inactive. This resulted in a moderately balanced label distribution, with 1358:1098 molecules in each bin, respectively. RDKit Descriptor Calculation Node from RDKit KNIME Integration (3.4.0) was used to generate 117 physicochemical descriptors for each of the modulators, which were then normalized and used to construct three predictive models, namely, Gradient Boosted Trees, Naïve Bayesian, and Logistic Regression (full description provided in the Methods section). We also tested the performance of other ML models, including k-nearest neighbor (k-NN), support vector machines (SVM), and RF but elected not to include them in our model, for a number of different reasons—k-NN and SVM both were found to predict a large number of positives in downstream applications (∼25% of the natural product data set), positive predictions made by k-NN were clustered in the small chemical space, whereas the performance of RF was found to be slightly worse than that of GBT and we opted to include only one decision tree-based model in our panel. We chose to use physiochemical descriptors rather than structural fingerprints so to minimize structural biases—as natural products cover a chemical space different from that of inhibitors resulting from medicinal chemistry campaigns, and biasing our models toward particular chemical moieties was not desirable. We built these models with the aim to screen large numbers of molecules; thus, it was paramount to minimize the number of false positives. As such, the key metric we were optimizing these models for was TNR (definition in the Methods section). To further minimize the false-positive rate, only molecules predicted to be active by the plurality of models were considered as positive predictions, i.e., akin to a jury approach. Another metric we aimed to keep as high as possible was the positive prediction value to ensure that the model can identify true positives while retaining good selectivity.

The three individual models and the consensus model (a model that considers a molecule positive only if the three individual models consider the molecule positive) were tuned and evaluated via 10-fold cross-validation. Briefly, the set of 2456 modulators was split 10 times into 9:1 training/validation subsets and used to train the models, with the resulting models then evaluated as described above. To scrutinize individual ML models, we plotted receiver operating characteristic (ROC) curves and calculated the area under curve (AUC) metric. For all the models, the AUC value was found to be well over 0.5, which shows that they are providing meaningful, nontrivial predictions (Figure S1a). Next, we calculated several different metrics for the individual and consensus models. We found that the consensus model was very good at differentiating true negatives from false positives, markedly better than any individual model (TNR = 0.89 for the consensus model, compared with 0.72, 0.52, and 0.71 for GBT, LR, and NB, respectively; full metrics for all the models can be found in Table S1). The same holds true for distinguishing true positives from false positives (PPV = 0.82 for the consensus model, compared with 0.78, 0.71, and 0.68 for GBT, LR, and NB, respectively).

As an additional control, we randomized the target label in the modulator data set and used it to build new ML models, with the expectation that these models will not result in meaningful predictions as label randomization breaks the link between biophysical information and modulatory activity. Indeed, the MCC for all three models was close to 0 (0.03, −0.01, −0.01, and 0.02 for GBT, LR, NB, and consensus, respectively), implying random predictions. The nonrandomized consensus model outperformed the randomized model according to all the metrics tested (TNR = 0.89 and 0.79, PPV = 0.82 and 0.58, MCC = 0.34 and 0.02 for the consensus prediction, and the consensus prediction with the shuffled label, respectively), showing that our models indeed provide meaningful outputs when trained on nonrandomized data.

### 5-LO Modulator Fragment Analysis

**Figure 2:**
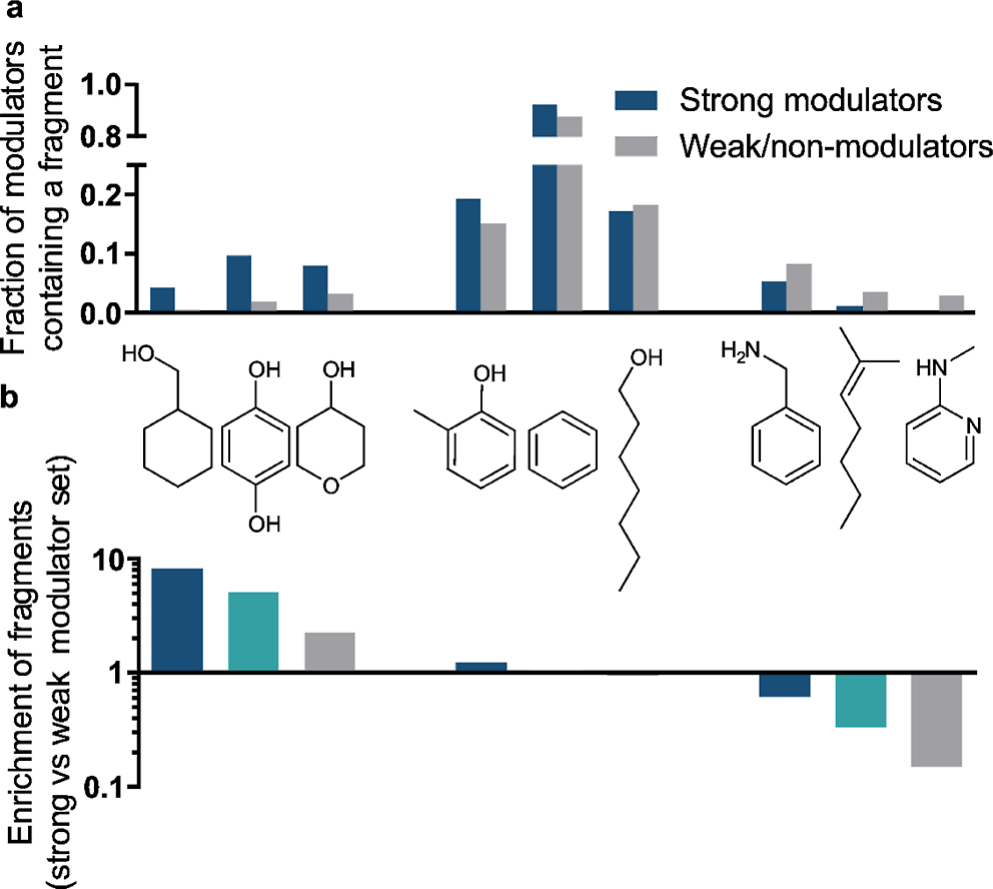
Examples of molecular fragments and their representations in the data sets of strong and weak 5-LO modulators. (a) Frequency of selected fragments in strong and weak 5-LO modulator data sets. (b) Enrichment of selected fragments in the strong modulator data set. Values above 1 correspond to the fragment being more common in the strong modulator data set compared to the weak modulator data set, and values below 1 imply the contrary.

Before using the implemented ML models to select suitable natural products for further testing, we aimed at better understanding the molecular features that might influence their ability to modulate the 5-LO pathway. For this aim, we carried out a motif analysis on the known 5-LO inhibitors. Briefly, we counted the occurrence of all the fragments sized 5–9 atoms from the strong and weak modulator data sets. Using that, we calculated the frequency of each fragment that corresponds to the fraction of molecules in a data set containing a particular fragment (Figure 2a and Table S2). In addition, we calculated the ratio of frequencies for the strong versus weak data sets; this ratio shows which molecular fragments are overrepresented in the strong 5-LO inhibitor data set and thus might play a role in 5-LO modulation (Figure 2b). Notably, we found that moieties known to affect ROS levels were overrepresented in the strong inhibitor data set (e.g., paraquinones). This corroborates findings presented in the literature—as 5-LO is an iron-dependent enzyme, redox cyclers can interfere with its oxidation state, thus reducing its activity.^14^

### Using a Data Set of FLAP Inhibitors to Build Models for the Discovery of 5-LO Pathway Modulators

FLAP is a nuclear membrane-bound protein, which assists 5-LO in product formation in intact cells by facilitating the access of 5-LO to its substrate AA.^31^ Upon inspecting the data set of FLAP inhibitors in ChEMBL, we have noticed a similar trend as that for 5-LO—a sizable part of reported FLAP inhibitors were determined via cell-based 5-LO assay, which screens for FLAP-dependent 5-LO product formation. Thus, we utilized our approach on this data set with the aim of finding 5-LO pathway modulators.

To implement predictive ML models for FLAP, we took an identical approach to the one with 5-LO. The FLAP inhibitor data set was collected and cleaned the same way as the 5-LO data set, leading to 2245 molecules being used for building FLAP models. Notably, the activity labels in this set were imbalanced, with 92% (2068) of the molecules being classified as strong inhibitors (IC_50_/*K*_D_/*K*_i_ < 1 μM), whereas this is the case for 55% of molecules in the 5-LO modulator data set. As such, we used BA as the metric to evaluate the resulting models as it is better suited for imbalanced data sets. LTA_4_ hydrolase, LTC_4_ synthase, and cPL-A_2_, other members of the 5-LO pathway, have much lower numbers of reported inhibitors with less chemical diversity; thus, we decided to focus solely on FLAP.

Using the FLAP modulator data set, we have built GBT, LR, and NB models. All had ROC AUC metrics above 0.5; thus, their predictions were nontrivial (Figure S1b). Again, the consensus model had superior metrics compared to individual models (BA = 0.67, 0.57, 0.54, and 0.65 for consensus, GBT, LR, and NB models, respectively, with full metrics in Table S1). As a control, we randomized the activity label while maintaining the class imbalance. As expected, these shuffled-data models could not predict the target class (MCC = 0.01, −0.01, 0.06, and −0.01 for consensus, GBT, LR, and NB models; MCC = 0.18 for the consensus FLAP model).

### Screening of Predicted 5-LO Modulators

**Figure 3:**
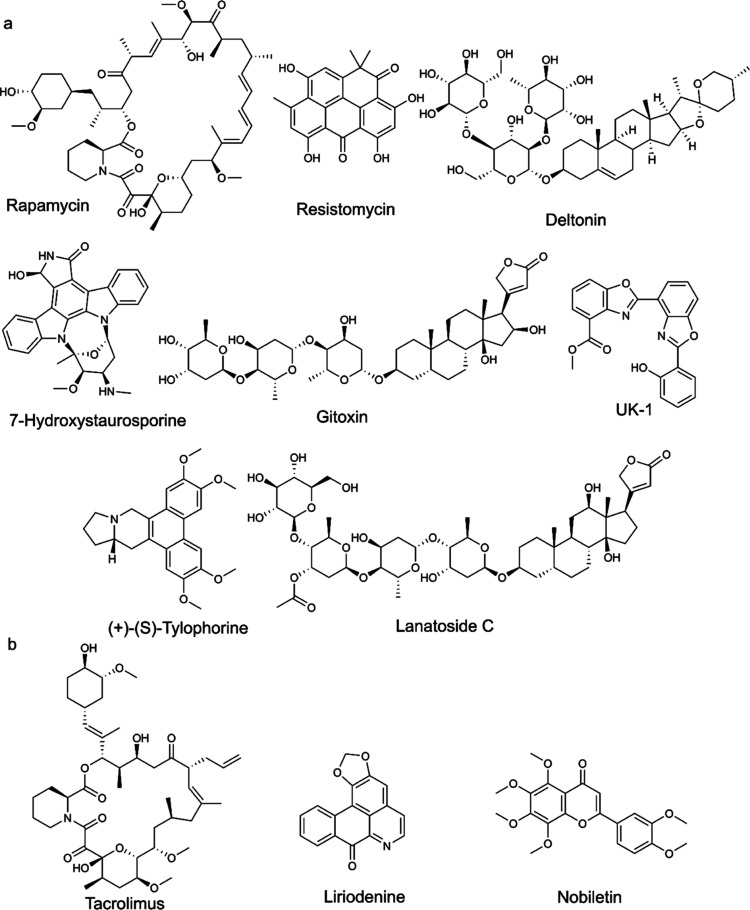
Natural products chosen for biological testing as 5-LO pathway modulators. (a) Eight natural products predicted to be active against 5-LO by all three predictive AI models. (b) Three natural products predicted to be active by two AI models.

With the models to predict 5-LO modulators and the motif analysis at hand, we went on to predict 5-LO pathway modulatory capabilities on the data set of 24,500 natural products and their derivatives. 1245 molecules were predicted as active by all three ML models (Table S3). To further triage the list, we investigated known biological activities of these molecules in the PubChem database (v. 1.5) and chosen molecules with profiles that could arise from 5-LO inhibition. We looked out for properties that would be expected of 5-LO inhibitors, e.g., anti-inflammatory, antiarthritic, and antiallergic properties as well as growth inhibition of cancer cell lines sensitive to 5-LO depletion. Based on these predictions, we have chosen eight molecules from the pool of natural products predicted to be 5-LO modulators by all three models as well as three molecules predicted to be positive by two models (Figure 3a,b).

As our aim was to use the ML models to predict and test the novel 5-LO inhibitor space, we needed to be sure that the chosen molecules were structurally disparate from those already tested against 5-LO and used to build the ML models. To investigate this, we carried out a similarity search between the molecules chosen for testing and 5-LO modulator data set, so as to find the most similar molecules between the two sets. Briefly, we converted the molecules into two different kinds of fingerprints and conducted a Tanimoto similarity search. Molecules found to be the most similar to chosen natural products and corresponding Tanimoto coefficients are reported in Table S4. Importantly, only one of our chosen molecules was found to have a scaffold similar to an already tested molecule—the flavone nobiletin. This demonstrates that the ML models used to predict 5-LO modulators are indeed capable of exploring previously untested chemical space.

**Figure 4:**
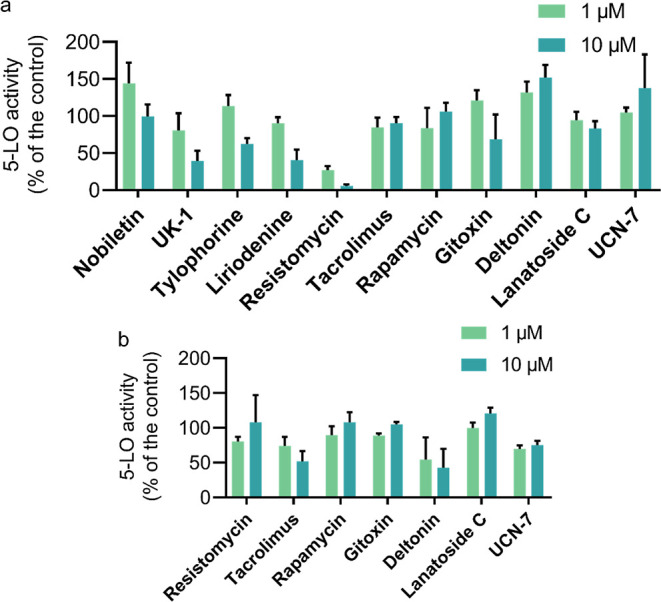
Screening results for 12 natural products as 5-LO pathway modulators. (a) Ability of natural products to inhibit the activity of 5-LO directly in a cell-free assay, as inferred from suppressing 5-LO product (LTB_4_ trans-isomers and 5-HETE) formation, with natural product concentrations in μM. (b) Ability of natural products to inhibit 5-LO product (LTB_4_, its trans-isomers, and 5-HETE) formation in the PMNL assay, with natural product concentrations in μM. Data are given as means + S.D., *n* = 3.

For the initial assessment of activity of the 11 molecules, we carried out two assays—inhibition of isolated 5-LO, which reports whether a molecule is a direct 5-LO enzyme inhibitor, and inhibition of 5-LO in Ca^2+^-ionophore A23187-activated PMNLs, which reports both direct 5-LO enzyme and 5-LO pathway modulators. As a reference drug, the 5-LO inhibitor zileuton (3 μM) was used, as reported before,^32^ and this drug blocked 5-LO activity in both test systems as expected (not shown). For the initial assessment, we tested two concentrations for each molecule −1 and 10 μM. Most of the molecules tested had some effect on 5-LO with at least two of them appearing to inhibit 5-LO directly, with IC_50_ values in the nanomolar range—resistomycin in the direct enzyme inhibition assay and deltonin in the cell-based PMNL assay (Figure 4a,b). Importantly, neither of the molecules are similar to the ones previously tested against 5-LO, so they constitute the novel 5-LO modulator chemical space (Table S4). Resistomycin’s inability to inhibit 5-LO product formation in PMNL could arise from its inability to cross cellular membranes due to the potential negative charge (its predicted p*K*_a_ values are 6.66, 7.58, 8.11, and 8.73, suggesting negative charge under physiological conditions). Conversely, deltonin was only active in the cell-based PMNL assay, which suggests that it is a 5-LO pathway modulator; thus, we investigated it further.

Similarly, we used the ML models made using the FLAP data set to predict 5-LO pathway modulators. This resulted in 940 positive predictions by the consensus model, similar to 1245 for 5-LO (Table S5). We have chosen 10 molecules for further testing based on their known activities, which we investigated in the PubChem database (Figure S2a). Chemical similarity search has again demonstrated that the molecules constitute novel chemical space compared with known 5-LO modulators, with pentacyclic triterpenes being the only overlapping scaffolds (Table S4). For initial screening, these compounds were tested either in an assay with isolated 5-LO or by using A23187-activated PMNL to study the modulation of cellular 5-LO product formation; the FLAP antagonist MK886 (0.3 μM) was used, as reported before.^33^ Intriguingly, three of the compounds—rhodomyrtone A, tipranavir, and bevirimat—were found to be potent inhibitors of 5-LO product synthesis in the PMNL assay with reduced efficiency against the 5-LO enzyme under cell-free conditions, which is consistent with the inhibition of the 5-LO/FLAP interaction (Figure S2b,c). Another compound, manzamine A, was found to inhibit 5-LO directly but not in the intact PMNL assay (Figure S2b,c).

We further investigated whether these compounds may act as disruptors of the 5-LO–FLAP interaction. In the cellular context, FLAP is required for the biosynthesis of 5-LO products from an endogenously released substrate; however, it is not necessary when 5-LO converts exogenous AA under cell-free conditions.^31^ Thus, if a molecule interferes with 5-LO-FLAP interactions, this inhibition can be partially overcome by providing cells with an exogenous substrate. We carried out concentration–response experiments using the PMNL assay for the three FLAP data set compounds active in both PMNL and direct 5-LO assays, either in the absence or presence of exogenous AA (Figure S2d). Interestingly, only one of the three compounds—bevirimat—partially lost its 5-LO inhibitory activity in the presence of AA, whereas the other two molecules became more potent. Moreover, bevirimat was found to be the strongest direct 5-LO inhibitor; therefore, in principle, it could interfere with both 5-LO catalytic activity and the 5-LO-FLAP interaction.

### Deltonin Does Not Permeabilize Cells Akin to Digitonin

**Figure 5:**
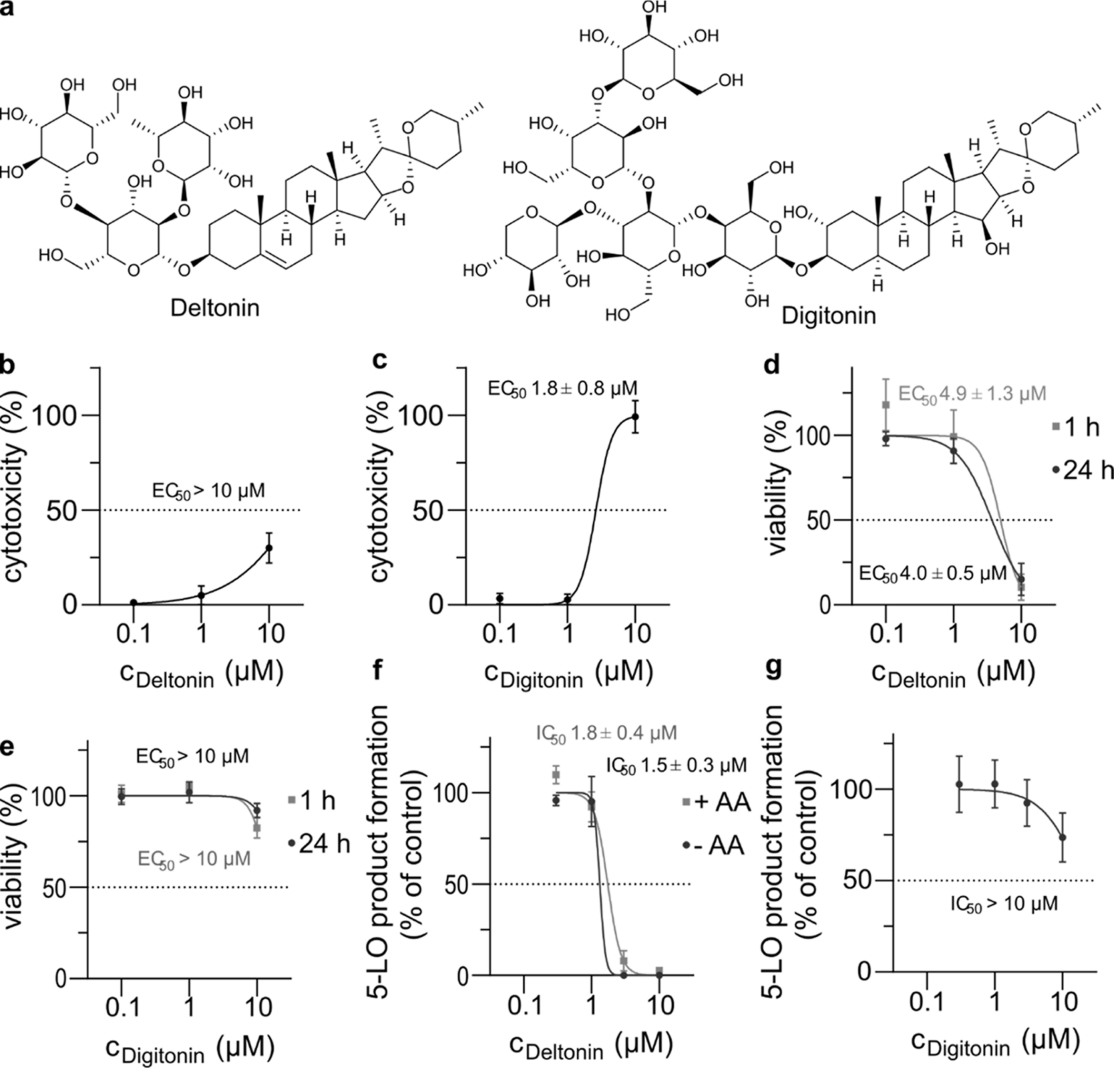
Deltonin and digitonin belong to the saponin family of natural products but act differentially on PMNL and PBMC. (a) Structures of deltonin and digitonin. (b) LDH assays were performed to evaluate the deltonin-induced cell lysis of PMNL. (c) LDH assays to evaluate digitonin-induced cell lysis of PMNL. (d) Results of an MTT assay to evaluate the cytotoxicity of deltonin toward PBMC over 1 and 24 h. (e) Results of an MTT assay to evaluate the cytotoxicity of digitonin toward PBMC over 1 and 24 h. (f) Concentration-dependent inhibition of 5-LO in a PMNL assay by deltonin, as inferred from the 5-LO product (LTB_4_, its trans-isomers, and 5-HETE) formation, with or without supplementation of exogenous arachidonic acid (AA). (g) Concentration-dependent inhibition of 5-LO in a PMNL assay by digitonin as inferred from 5-LO product (LTB_4_, its trans-isomers, and 5-HETE) formation. Data are means ± S.D., *n* = 3.

Deltonin is a saponin; thus, we decided to compare its bioactivity with another molecule from the same family of natural products, digitonin. The alkaloid parts in these two molecules differ only by a double bond and a couple of hydroxyl groups, with the major difference between these molecules being the glycoside part (Figure 5a). Digitonin permeabilizes cellular membranes;^34^ thus, we explored the possibility of deltonin having similar features. First, we tested the ability of these compounds to induce the lysis of PMNL. For this aim, we have carried out an LDH assay, in which we treated PMNL with one of the two saponins for 20 min. This was followed by quantification of extracellular LDH that leaks from cells with compromised membranes to evaluate the extent of cell lysis. We found that deltonin can have some effect on the integrity of cellular membranes at the highest concentration tested (10 μM), although this might be a result of its cytotoxicity. Digitonin, on the other hand, induced a complete lysis of the cells at the same concentration, in line with reports by others.^34^ To gain further insight into deltonin’s mode of action, we carried out a hemolysis assay. Briefly, we incubated deltonin with sheep blood for 24 h, followed by quantification of the released hemoglobin. Some lysis was detected with 10 μM deltonin, potentially a result of its cytotoxicity, which echoes the results of LDH assay (Figure S3). As discussed previously, deltonin can extensively inhibit the activity of 5-LO at lower concentrations; thus, cell lysis cannot be the mechanism behind this phenomenon.

Next, we investigated the short- and long-term cytotoxicity of deltonin and digitonin. For this aim, we treated PBMC with one of the two saponins for 1 or 24 h (PMNL are too short-lived for this assay analyzing effects at 24 h). We found that deltonin exhibits potent cytotoxicity, with an estimated EC_50_ value of 4.0 μM after 24 h treatment, in contrast with digitonin that was hardly active (Figure 5d,e). Interestingly, little difference was observed between 1 and 24 h treatment for both saponins, indicating a mechanism of action faster than the timeframes tested. Altogether, this corroborates that the observed deltonin-induced lysis is likely a result of its cytotoxicity whereas digitonin permeabilizes cells; thus, the two compounds have different mechanisms of action despite similarities in structure.

### Deltonin Inhibits 5-LO Product Formation in PMNL but Not in M1 Macrophages

We went on to establish an EC_50_ value against 5-LO for both compounds in the PMNL assay format as well as to investigate whether inhibition by deltonin could be reversed by carrying out the assay with exogenously added 5-LO substrate AA. Deltonin was found to inhibit 5-LO product formation with an IC_50_ value of 1.5 μM, with exogenous AA (20 μM) having little influence on the inhibitory potency (Figure 5f). This suggests that 5-LO inhibition is not a result of substrate depletion (due to cPLA_2_ inhibition) or delivery inhibition (by blocking FLAP), as otherwise, AA would reverse it. Digitonin was found to somewhat affect 5-LO product biosynthesis at higher concentrations, likely as a result of compromising cell membranes (Figure 5g).

**Figure 6:**
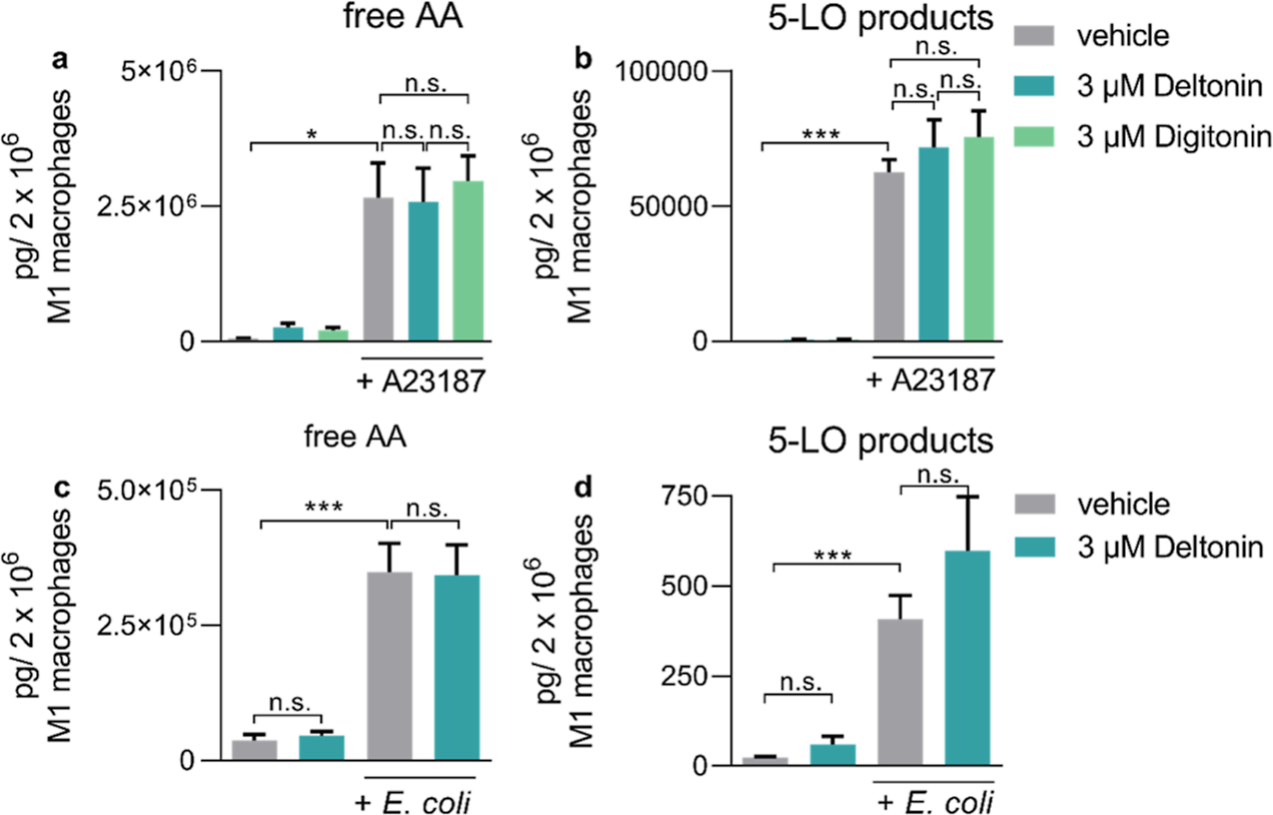
Treatment of activated M1 macrophages with deltonin or digitonin does not modulate 5-LO activity or substrate availability. (a) Effects of deltonin or digitonin on AA release in A23187-stimulated M1 macrophages (*n* = 3). (b) Effects of deltonin or digitonin on the formation of 5-LO products (LTB_4_, its trans-isomers, and 5-HETE) in A23187-stimulated M1 macrophages (*n* = 3). (c) Effects of deltonin or digitonin on the release of AA in *E. coli*-stimulated M1 macrophages (*n* = 4). (d) Treatment with deltonin or digitonin does not modulate the formation of 5-LO products in *E. coli*-stimulated M1 macrophages (*n* = 4). Data are means + S.D.; n.s. = not significant, *p < 0.05, ***p < 0.001.

The 5-LO pathway is also expressed in other innate immune cells, e.g., M1 macrophages. Thus, we investigated whether treatment of human monocyte-derived M1 macrophages with deltonin could affect the formation of 5-LO products in these cells. To activate the M1 macrophages, we utilized either stimulation with A23187 or pathogenic *E. coli*. In both cases, treatment with deltonin had no significant effects (Figure 6a–d), in contrast to the 5-LO inhibitor zileuton that blocked 5-LO product formation in these cells under the same conditions.^28^ Like in PMNL, also in M1 macrophages, digitonin failed to effectively inhibit the 5-LO product formation. Additionally, we tested whether deltonin could affect the release of AA and other polyunsaturated fatty acids (PUFAs) and the activity of enzymes involved in the formation of additional inflammation-related lipid mediators in M1 macrophages, such as COX-1/2, 12-LO, and 15-LO (Figure S4a–h). Compared to 5-LO, we did not observe significant changes in the levels of various lipid mediators by these enzymes. Altogether, this demonstrates that deltonin modulates 5-LO and lipid signaling in a cell type-selective manner, being a potent 5-LO pathway modulator in PMNL, devoid of efficacy in M1 macrophages.

### Deltonin Elevates Intracellular Ca^2+^ Levels in PMNL

5-LO and cPLA_2_ activities are stimulated by Ca^2+^, which is why Ca^2+^ ionophores can be used to induce 5-LO product formation in intact cells.^35,36^ On the other hand, exposure of 5-LO to Ca^2+^ in the absence of AA (or other PUFAs as substrates) can inactivate the enzyme in a time-dependent manner due to complex redox-dependent mechanisms affecting the catalytic cycle of the active-site iron.^37−39^ For example, sphingosine-1-phosphate induced Ca^2+^ mobilization in PMNL and thereby led to irreversible inactivation of 5-LO seemingly involving ROS. Thus, we investigated if deltonin and digitonin could affect intracellular Ca^2+^ levels in PMNL. For this aim, we utilized Fura-2-AM, a ratiometric Ca^2+^ indicator in intact cells. As positive control, we used fMLP, a peptide known to elevate intracellular Ca^2+^ and to activate macrophages, because unlike many Ca^2+^ ionophores (e.g., A23187), it does not interfere with fluorescent probes that we used in these assays.^40^

**Figure 7:**
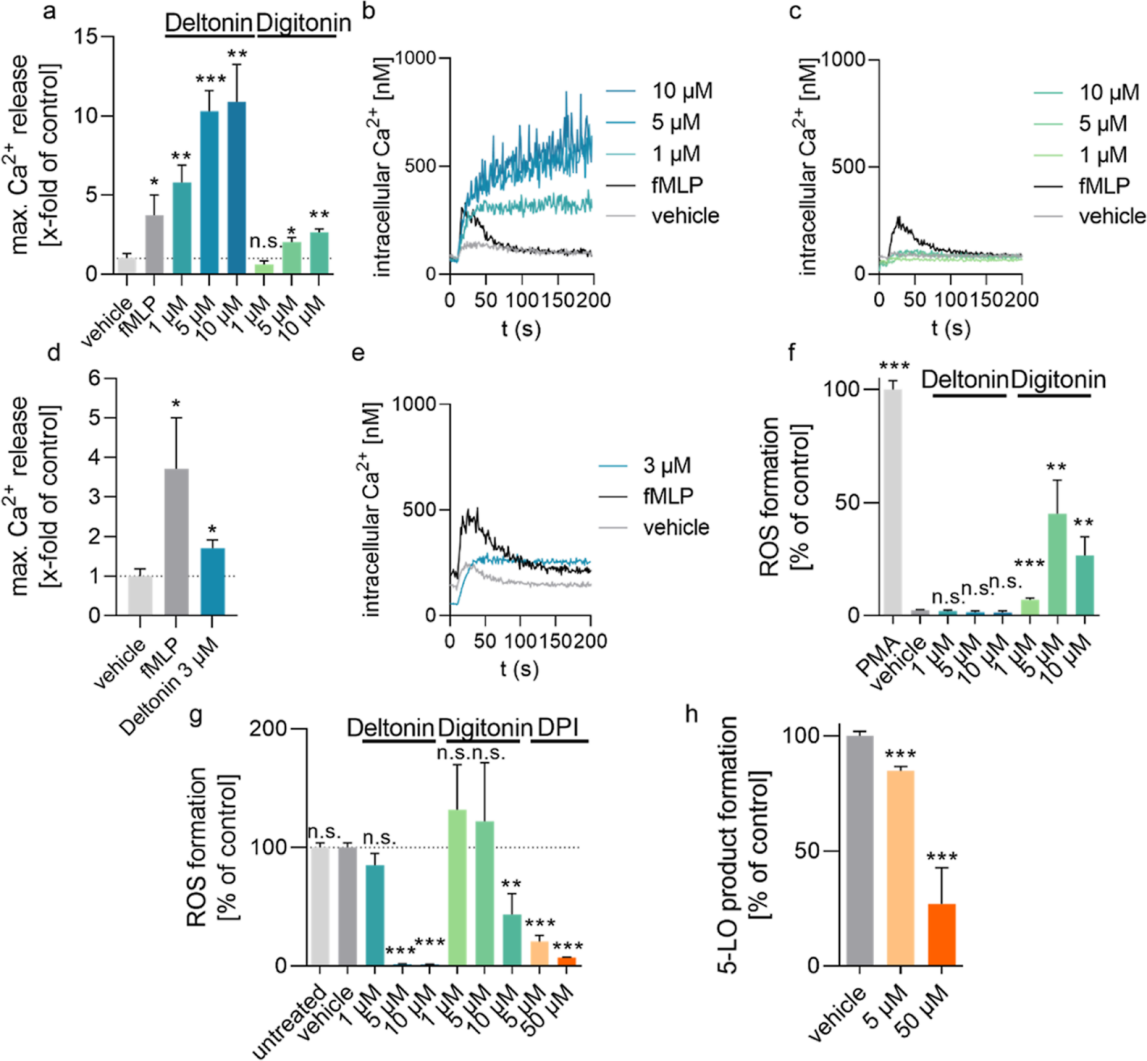
Deltonin potently modulates Ca^2+^influx and ROS levels in PMNL. (a) Maximal calcium levels in PMNL upon treatment with fMLP (1 μM), deltonin, or digitonin (*n* = 3). (b) Real-time measurement of Ca^2+^ levels in PMNL upon treatment with fMLP or deltonin (*n* = 3, representative graph shown). (c) Real-time measurement of Ca^2+^ levels in PMNL upon treatment with fMLP or digitonin (*n* = 3, representative graph shown). (d) Maximal Ca^2+^ levels in M1 macrophages upon treatment with fMLP or deltonin (*n* = 3). (e) Real-time measurement of Ca^2+^ levels in M1 macrophages upon treatment with fMLP or deltonin (*n* = 3, representative graph shown). (f) Levels of ROS in PMNL stained with DCFH-DA (2 μg/mL) upon treatment with deltonin and digitonin (*n* = 3). (g) Effect of deltonin, digitonin, and DPI on ROS formation on PMNL treated with PMA (1 μM) (*n* = 3). (h) Effect of DPI on 5-LO product (LTB_4_ and its trans-isomers, 5-HETE) formation in PMNL (*n* = 3). Data are given as means + S.D.; n.s. = not significant, *p < 0.05, **p < 0.01, and ***p < 0.001 when compared to the vehicle control.

We measured temporal intracellular Ca^2+^ levels up to 200 s, after addition of vehicle, fMLP, deltonin, or digitonin. fMLP caused a rapid Ca^2+^ influx, with the effect dissipating over 100 s and reaching the base level. Deltonin also led to a rapid increase in Ca^2+^, but unlike fMLP, the increased Ca^2+^ levels remained elevated throughout the course of the experiment (Figure 7a,b). The effect of deltonin was concentration-dependent—the maximal elevation of Ca^2+^ was similar to one achieved with fMLP (both at 1 μM), but higher levels were reached with deltonin at 5 and 10 μM. Digitonin also led to an increase in intracellular Ca^2+^ at higher concentrations but much less pronounced, even when 10 μM was used; the effect was smaller than with fMLP at 1 μM (Figure 7a,b). This demonstrates that deltonin potently elevates Ca^2+^ levels in PMNL, which might contribute to the 5-LO inhibitory activity.

If modulation of Ca^2+^ levels indeed underlies deltonin-induced 5-LO inhibition in PMNL, Ca^2+^ modulation by deltonin should be less pronounced in M1 macrophages as it does not inhibit 5-LO product formation in these cells. Thus, we carried out real-time Ca^2+^ measurements upon treating M1 macrophages with fMLP or deltonin. The known macrophage activator fMLP induced a strong elevation of intracellular Ca^2+^, as expected (Figure 7d,e). Deltonin treatment led to a modest increase in Ca^2+^ levels, albeit a much smaller one when compared with its effect on PMNL or treatment of M1 macrophages with 1 μM fMLP under the same conditions. This further supports the hypothesis that deltonin-induced 5-LO inactivation occurs through Ca^2+^ modulation, as treatment with deltonin affects both processes more in PMNL than in M1 macrophages.

### Deltonin Is a Redox Modulator

The activity of 5-LO depends on the redox environment in the cell. 5-LO is a ferroprotein, with its catalytic cycle initiated by the oxidation of Fe^2+^ into Fe^3+^, a process initiated by lipid hydroperoxides.^14^ Thus, impaired cellular ROS levels can prevent the 5-LO product formation. We measured the ability of deltonin and digitonin to affect ROS formation in PMNL by employing DCFH-DA, a redox-state-sensitive fluorescent probe that gets localized inside cells upon treatment. To investigate the ability of the tested molecules to induce ROS formation, we treated cells with PMA (phorbol 12-myristate 13-acetate) as a positive control and compared its ability to elevate ROS with the two saponins. PMA is a known activator of NADPH oxidase, which leads to the formation of ROS and subsequently lipid hydroperoxides.^41^ In this experiment, deltonin failed to induce ROS formation, whereas digitonin was active, although less pronounced than PMA (Figure 7f). To test whether the compounds can prevent PMA-induced ROS formation, we pretreated cells with one of the two compounds or the NADPH oxidase inhibitor DPI (diphenyleneiodonium) that suppresses ROS formation, followed by stimulation with PMA. Strikingly, deltonin completely abolished ROS formation at 5 and 10 μM with a milder effect at 1 μM, in line with its effects on 5-LO inhibition (Figure 7g). 10 μM of digitonin also led to reduced ROS levels but to a lesser extent than deltonin. DPI led to a dramatic reduction of ROS levels at the two tested concentrations, albeit it was not able to completely suppress ROS levels akin to deltonin. Interestingly, we found that in addition to reducing ROS levels, treatment with DPI also led to reduced 5-LO product formation in PMNL which provides further evidence that impairment of the cellular redox tone can be linked to 5-LO product formation (Figure 7h). Overall, this demonstrates that deltonin could be inhibiting the 5-LO pathway in PMNL through reduction of cellular ROS levels, preventing the activation of the 5-LO enzyme.

## Conclusions

We have built ML models to identify new modulators of the 5-LO pathway using a database of known 5-LO or FLAP inhibitors as the training set. Interestingly, the two training sets contained both direct 5-LO enzyme and pathway modulators, and as such, these models were able to detect both direct and indirect inhibitors. We applied these models on a data set of natural products and their derivatives, and after further investigating their properties in the PubChem database, we have chosen 12 and 10 natural products predicted to be active in the 5-LO and FLAP models, respectively. For both models, several tested molecules were found to be modulators of the 5-LO pathway: either direct 5-LO enzyme inhibitors or suppressors of 5-LO product formation in PMNL (five molecules from the 5-LO set and four molecules from the FLAP set inhibited at least 50% of 5-LO activity at one or two of the tested concentrations). Overall, it demonstrates that the described approach is fit for the purpose of elucidating new natural product–5-LO pathway interactions.

We then carried out an extensive mechanistic evaluation on one of the strongest discovered modulators, deltonin. The initial screen revealed this molecule to be a strong inhibitor in the cell-based PMNL assay, without direct inhibition of 5-LO. After testing several hypotheses, we found that deltonin affects both intracellular Ca^2+^ levels (elevation) and the redox state (suppression) of cells, both of which are determinants for cellular 5-LO product formation. Intriguingly, we found that deltonin potently affects 5-LO in PMNL but not in M1 macrophages; thus, this phenomenon is cell type-selective. Further studies are required to pinpoint more accurately which processes in the cell are affected by deltonin and to determine the basis of its cell type selectivity. Given the emerging emphasis on cell and tissue selectivity in therapeutics, understanding the mechanism through which deltonin achieves this selectivity can inform the design of novel medicines. We also tested whether natural products predicted to be active from a FLAP inhibitor data set could suppress cellular 5-LO product formation. Intriguingly, we identified four 5-LO modulators from these predictions, but only one of these molecules had an inhibitory profile that could be consistent with the disruption of 5-LO–FLAP interactions. This is likely a result of the training set containing information about both direct and indirect FLAP modulators. Since FLAP has no enzymatic or any other measurable bioactivity under cell-free conditions, analysis of cellular 5-LO product formation from an endogenous substrate is the only assessment of its functionality.

Together, this study provides a framework for how ML models may be used to elucidate targets and affected pathways of natural products or other small molecules as well as to investigate their biological mechanisms. The number of known natural products is exceedingly large for physical testing (e.g., SuperNatural III database reports 790,096 entries^42^), especially given their difficulty of isolation and limited availability. Natural products are rich in untapped chemical and biomechanistic diversity; hence, new ways to investigate their functions can lead to a better understanding of interactions between small molecules and biomolecules, with the potential to lead to new classes of therapeutics. As such, emerging in silico tools are well placed to probe the space of natural products and lead to new mechanistic and therapeutic insights.

## Supporting Information Available

Data set of 5-LO modulators used to build ML models, data set of commercially available natural products, data set of FLAP modulators used to build ML models, metrics of ML models used in this study, list of molecular fragments in 5-LO modulators and their frequencies in strong and weak modulator subsets, 5-LO modulator-derived ML model predictions on the natural product data set, similarity search of tested natural products in the 5-LO modulator data set, FLAP modulator-derived ML model predictions on the natural product data set, KNIME workflow used to build ML models, and KNIME workflow used to carry out 5-LO fragment analysis (PDF)
5-LO molecular data set, commercially available natural products, FLAP modular data set, ML model metrics, 5-LO modulator fragment analysis, predictions from the 5-LO data set, predictions from the FLAP data set, ML workflow, and fragment analysis workflow(ZIP)
