# Supplementary material for: Machine Learning Uncovers Natural Product Modulators of the 5-Lipoxygenase Pathway and Facilitates the Elucidation of Their Biological Mechanisms
Source: ACS Chem Biol. 2023 Dec 27;19(1):217–29. doi: 10.1021/acschembio.3c00725 (PMC10804367; doi:10.1021/acschembio.3c00725)
Supplement: Supplementary file 1 — cb3c00725_si_001.pdf [file cb3c00725_si_001.pdf]

Supporting Information

Machine learning uncovers natural product modulators of the 5-lipoxygenase pathway and facilitates elucidation of their biological mechanisms

Sigitas Mikutis[#*1], Stefanie Lawrinowitz[#2], Christian Kretzer[2], Lavinia Dunsmore[1], Laurynas Sketeris[1], Tiago Rodrigues[3], Oliver Werz*[2] & Gonçalo J. L. Bernardes*[1,4]

[1]Yusuf Hamied Department of Chemistry, University of Cambridge, Lensfield Road, Cambridge CB2 1EW, UK

[2]Department of Pharmaceutical/Medicinal Chemistry, Institute of Pharmacy, Friedrich Schiller University Jena, Philosophenweg 14, 07743 Jena, Germany

[3]Instituto de Investigação do Medicamento (iMed), Faculdade de Farmácia, Universidade de Lisboa, Av. Prof. Gama Pinto, 1649-003 Lisbon, Portugal

[4]Instituto de Medicina Molecular João Lobo Antunes, Faculdade de Medicina, Universidade de Lisboa, Avenida Professor Egas Moniz, 1649-028, Lisboa, Portugal

[#]These authors contributed equally to this work.

Correspondence should be addressed to G.J.L.B. or S.M. or O.W.:

E-mail: gb453@cam.ac.uk
E-mail: sm2029@cantab.ac.uk
E-mail: oliver.werz@uni-jena.de

Table of Contents

1. **Supporting Figures & Table** **S3**
2. **Methods** **S11**
3. **References** **S17**

Supporting Figures & Table

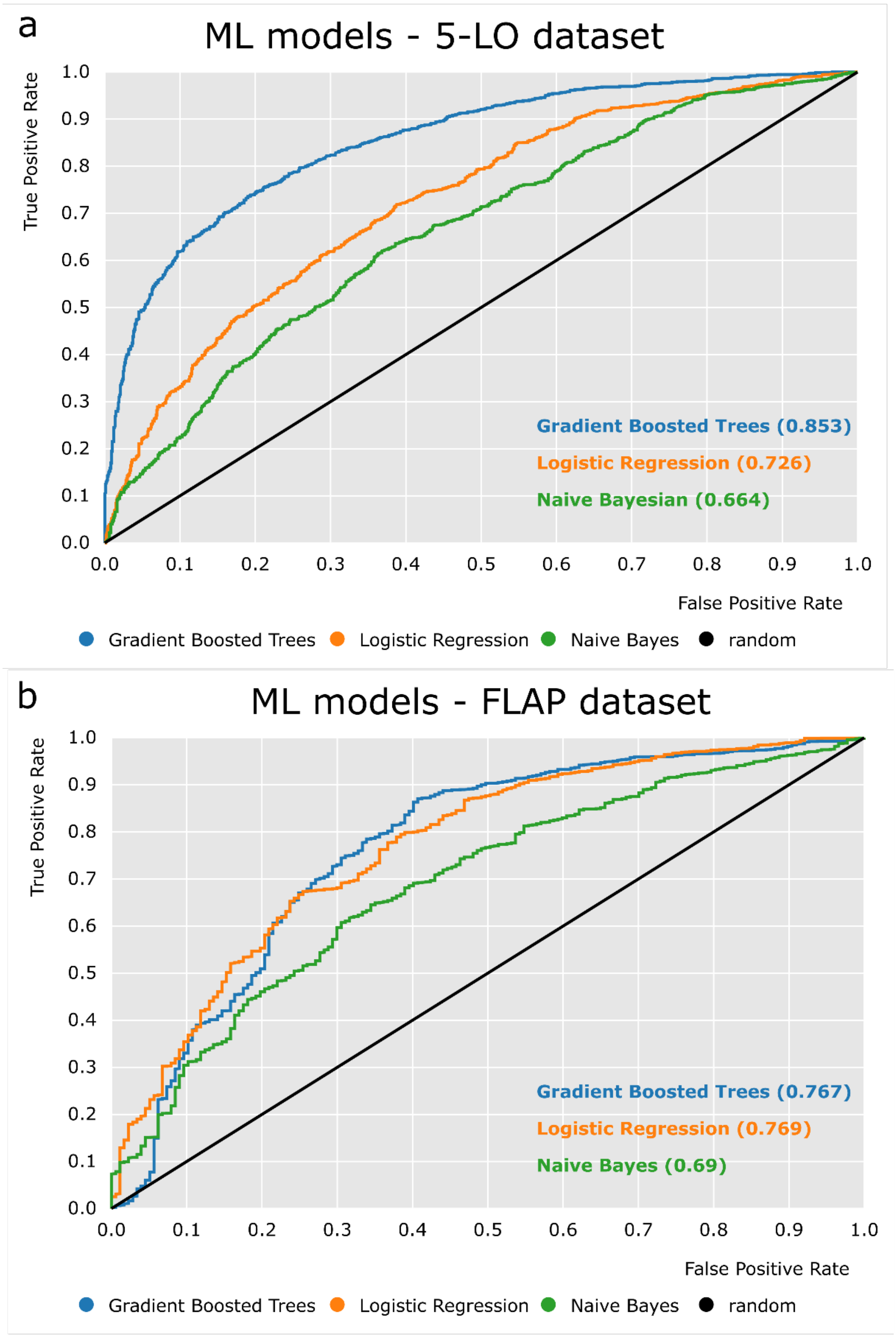

Figure S1 | **Receiver operating characteristic (ROC) curves and area under curve (AUC) metrics.** (a) ROC curves for machine learning models built from 5-LO dataset. (b) ROC curves for machine learning models built from FLAP dataset. AUC metrics are given in the bottom right corner.

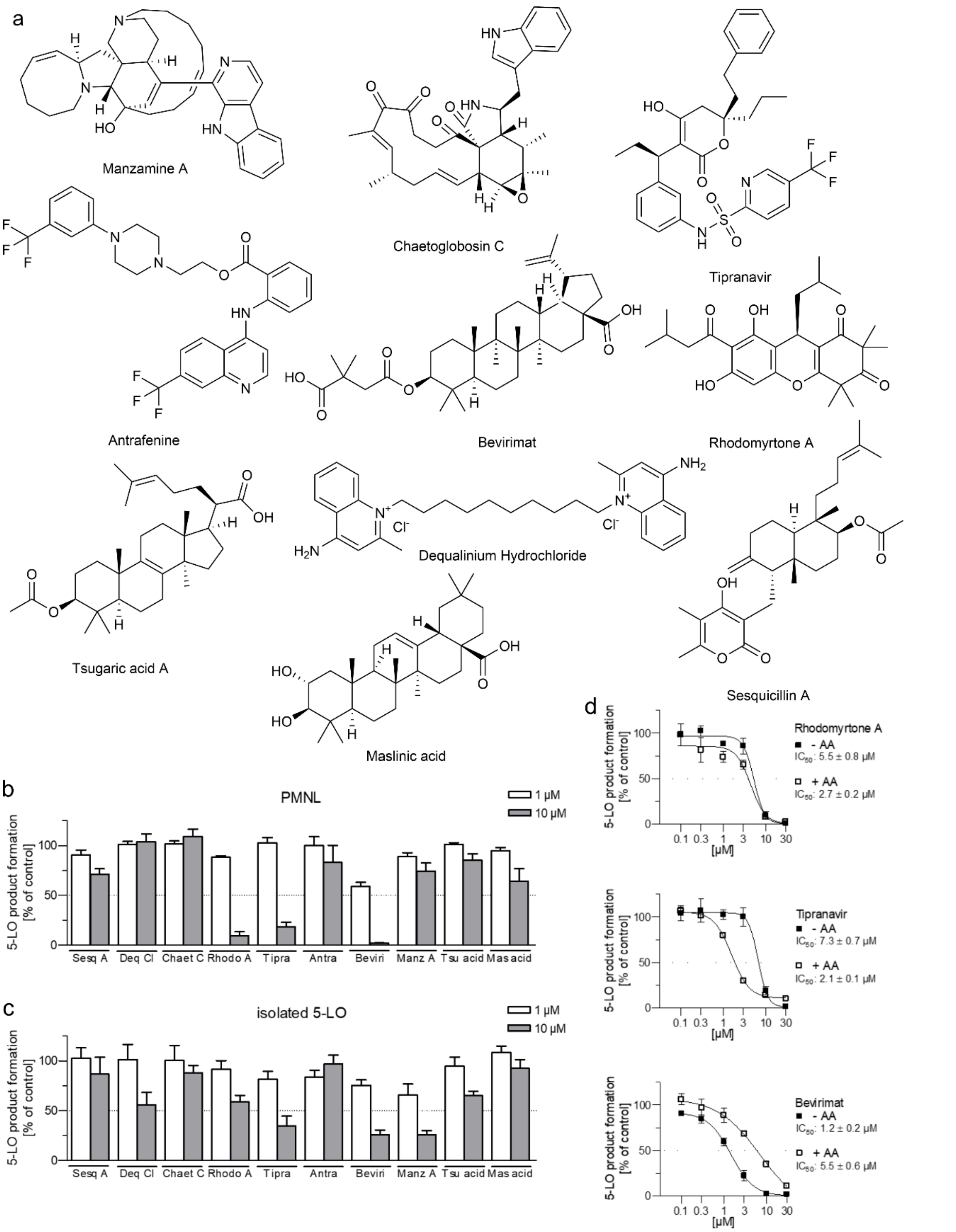

Figure S2 | **Natural products chosen through models built on FLAP inhibitors can modulate 5-LO levels.** (a) 10 natural products predicted to be positive by all 3 FLAP models chosen for further testing. (b) Their activities against 5-LO in PMNLs. (c) Their activities against isolated 5-LO. (d) Dose response curves, in presence and absence of AA.

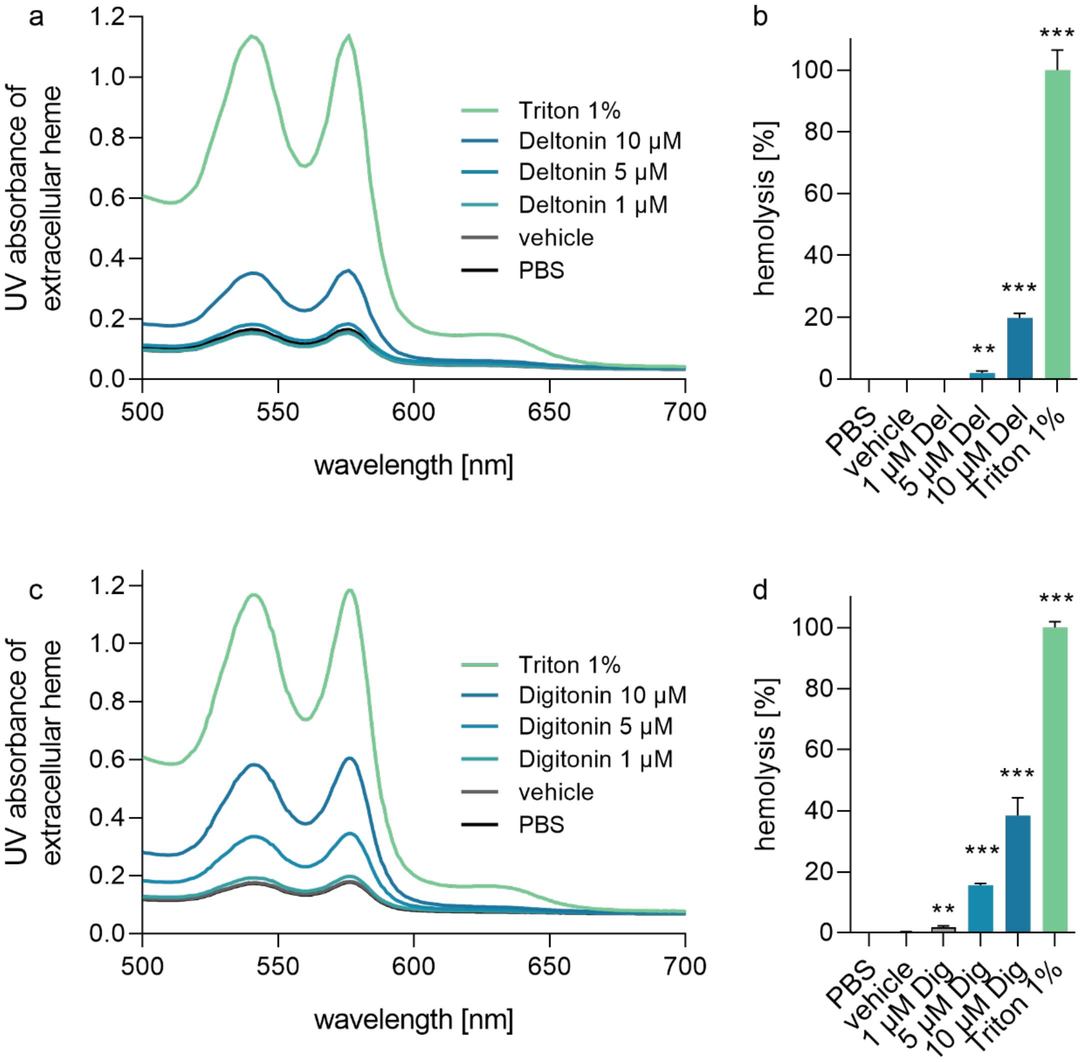

Figure S3 | **Investigation of deltonin's hemolytic ability in sheep's blood assay.** (a) An example of an absorbance spectrum resulting from sheep blood hemolysis after it has been treated with deltonin or a specified control for 24 hours. (b) Quantification of sheep blood hemolysis after it has been treated with deltonin or a specified control for 24 hours. n = 3, error bars correspond to standard deviation. (c) An example of an absorbance spectrum resulting from sheep blood hemolysis after it has been treated with digitonin or a specified control for 24 hours. (d) Quantification of sheep blood hemolysis after it has been treated with digitonin or a specified control for 24 hours. n = 3, error bars correspond to standard deviation. ** $p < 0.01$, *** $p < 0.001$ when compared to the vehicle control.

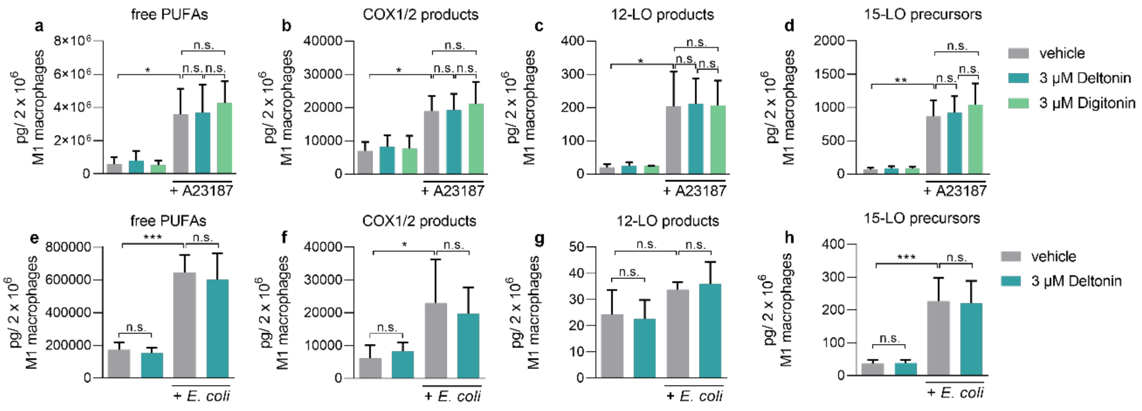

Figure S4. | **Treatment of activated M1 macrophages with deltonin or digitonin does not modulate lipid signalling pathways.** (a) Effects of treatment with deltonin or digitonin on the concentration of released PUFAs (AA, DHA, EPA, and α-Linolenic acid) in A23187-stimulated M1 macrophages (n = 3). (b) Effects of treatment with deltonin or digitonin on the concentration of COX1/2 products (11-HETE, 11-HEPE, 15-keto PGE_2, PGE_2, PGD_2, $PGF_{2\alpha}$, and TXB_2) in A23187-stimulated M1 macrophages (n = 3). (c) Effects of treatment with deltonin or digitonin on the concentration of 12-LO products (12-HETE and 12-HEPE) in A23187-stimulated M1 macrophages (n = 3). (d) Effects of treatment with deltonin or digitonin on the concentration of 15-LO products (15-HEPE, 15-HETE, 5,15-diHETE, and 5,15-diHEPE) in A23187-stimulated M1 macrophages (n = 3). (e) Effects of treatment with deltonin or digitonin on the concentration of released PUFAs (AA, DHA, EPA, and α-Linolenic acid) in human-pathogenic E.Coli-stimulated M1 macrophages (n = 4). (f) Effects of treatment with deltonin or digitonin on the concentration of COX1/2 products (11-HETE, 11-HEPE, 15-keto PGE_2, PGE_2, PGD_2, $PGF_{2\alpha}$, and TXB_2) in human-pathogenic E.Coli-stimulated M1 macrophages (n = 4). (g) Effects of treatment with deltonin or digitonin on the concentration of 12-LO products (12-HETE and 12-HEPE) in in human-pathogenic E.Coli-stimulated M1 macrophages (n = 4). (h) Effects of treatment with deltonin or digitonin on the concentration of 15-LO products (15-HEPE, 15-HETE, 5,15-diHETE, and 5,15-diHEPE) in in human-pathogenic E.Coli-stimulated M1 macrophages (n = 4). Error bars correspond to SD. n.s. = not significant, * $p < 0.05$, ** $p < 0.05$, *** $p < 0.001$.

Table S4 | **Similarity search of tested natural products in the 5-LO modulator dataset.** Numbers indicate Tanimoto similarity coefficient.

Tested molecule	The most similar molecule (RDKit fingerprint)	The most similar molecule (Atom pair fingerprint)
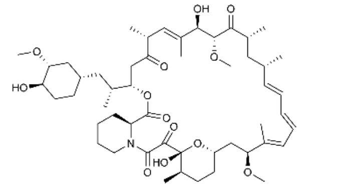 Rapamycin	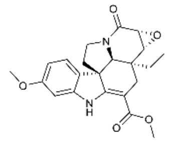 0.471	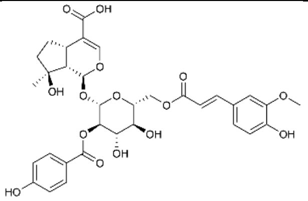 0.506
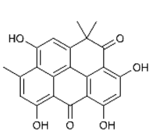 Resistomycin	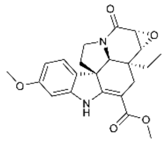 0.622	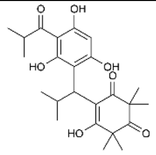 0.417
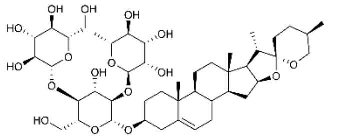 Deltonin	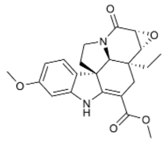 0.559	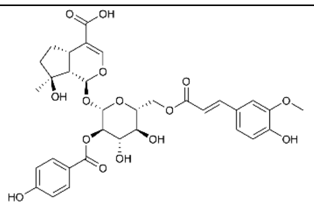 0.431
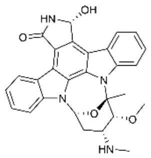 7ihydroxystaurosporine	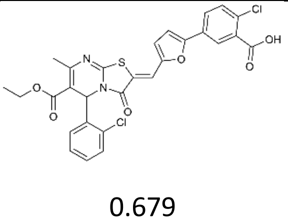 0.679	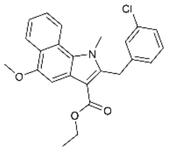 0.426
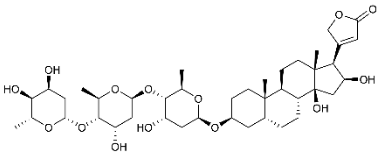 Gitoxin	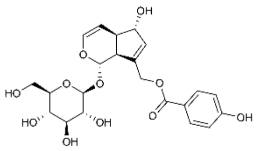 0.546	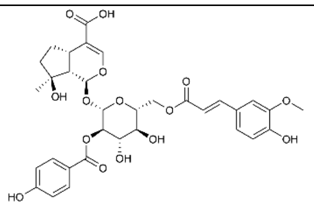 0.439
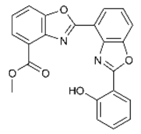 UK-1	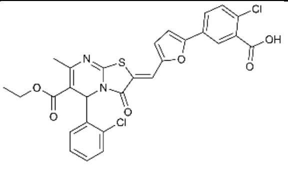 0.544	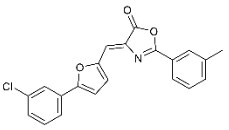 0.497

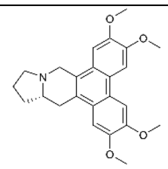 (+)-(S)-Tylophorine	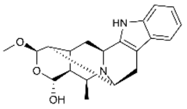 0.448	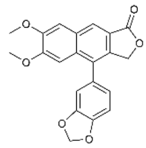 0.515
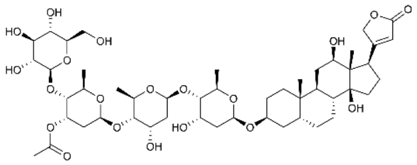 Lanatoside C	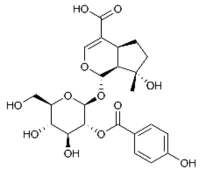 0.564	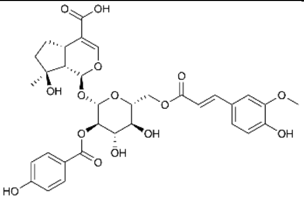 0.434
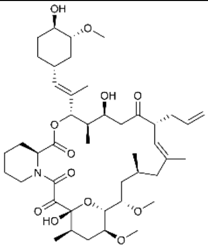 Tacrolimus	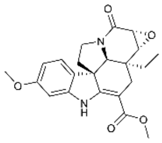 0.563	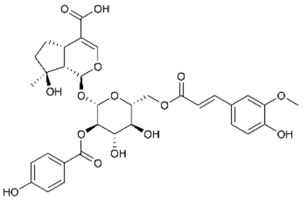 0.502
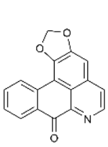 Liriodenine	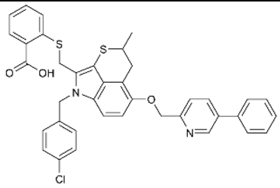 0.542	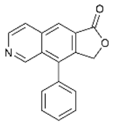 0.608
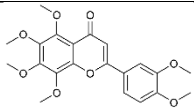 Nobiletin	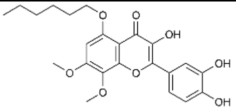 0.866	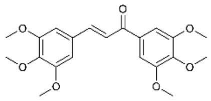 0.629
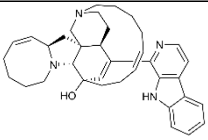 Manzamine A	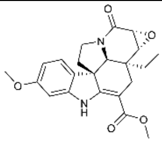 0.659	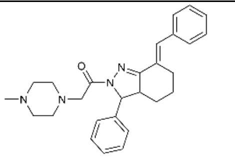 0.458
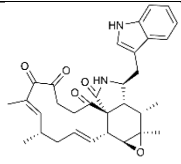 Chaetoglobosin C	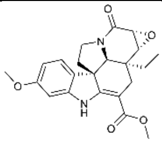 0.612	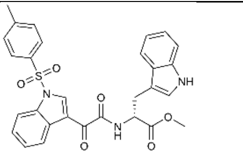 0.516

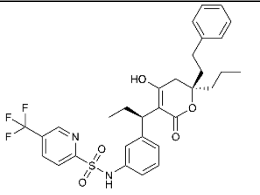 Tipranavir	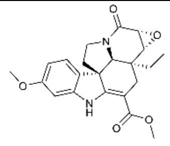 0.601	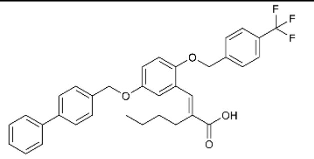 0.487
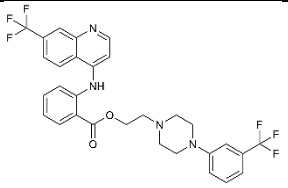 Antrafenine	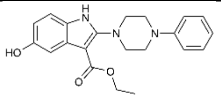 0.492	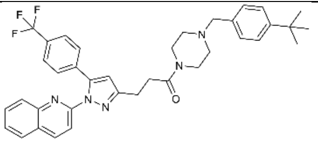 0.533
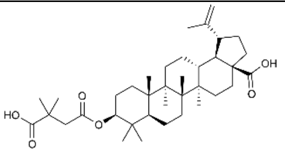 Bevirimat	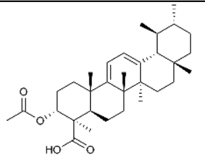 0.612	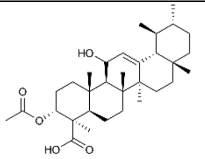 0.613
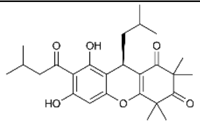 Rhodomyrtone A	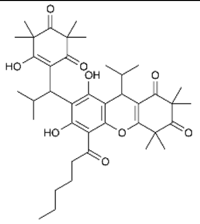 0.964	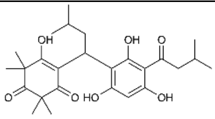 0.823
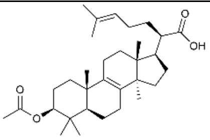 Tsugaric acid A	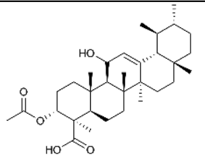 0.715	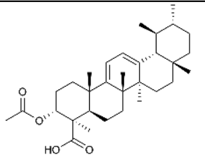 0.634
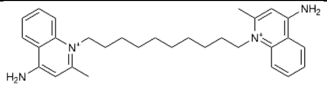 Dequalinium	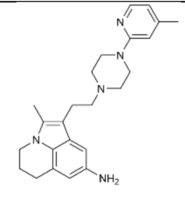 0.409	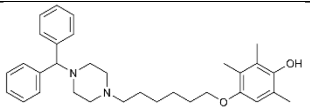 0.432
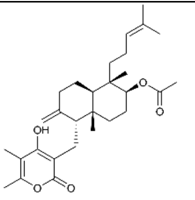	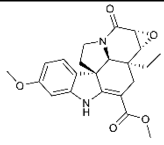 0.471	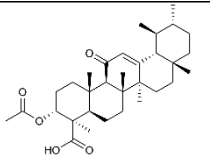 0.476

Sesquicillin A		
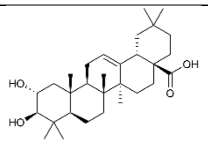<br>Maslinic acid	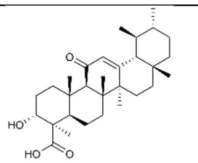<br>0.682	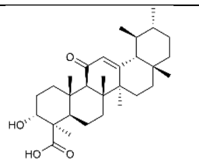<br>0.665

Methods

Data preparation. 5128 entries describing small molecule modulation of 5-LO and 4781 describing modulation of FLAP were downloaded from CHEMBL23 database an uploaded into KNIME analytics platform (v. 3.4.0)[1]. Only entries that report exact IC_{50}, K_D or K_i relationships at nanomolar concentrations were kept and duplicates were removed, leading to 2456 and 2245 entries for 5-LO and FLAP datasets. Molecules with reported activities below 1,000 nM were classified as strong modulators (label: 1) with the rest labelled as weak (label: 0). RDKit descriptors (117) were calculated for each molecule and were then normalised.

ML models. No code ML models were built with native nodes in the KNIME 3.4.0 environment, using the normalized descriptors of 2456 5-LO and 2245 FLAP modulators. A 10-fold cross-validation was performed to optimize hyperparameters and gauge the performance of three classifiers: gradient-boosted trees (GBT)[2], logistic regression (LR)[3] and naïve Bayesian (NB)[4] models, according to the True Negative Rate (TNR), Positive Predictive Value (PPV), the Matthew's Correlation Coefficient and Balanced Accuracy (BA)[5], defined as:

$$TNR = \frac{TN}{TN + FP} \quad (1)$$

$$PPV = \frac{TP}{TP + FP} \quad (2)$$

$$MCC = \frac{TP \times TN - FP \times FN}{\sqrt{(TP + FP)(TP + FN)(TN + FP)(TN + FN)}} \quad (3)$$

$$BA = \frac{1}{2}\left(\frac{TP}{TP + FN} + \frac{TN}{TN + FP}\right) \quad (4)$$

Where TP – number of true positives, TN – number of true negatives, FP – number of false positives and FN – number of false negatives. We use 1234 as random state. The relevant hyperparameters for the 5-LO models are as follows: GBT – tree depth = 4, number of trees = 500, learning rate = 0.05; LR – maximal number of epochs = 100, epsilon = 1.0E-05, step size = 0.1; NB – default probability = 0.0, maximum number of unique nominal values per attribute = 20. For FLAP models, the parameters are the same except GBT – learning rate = 0.15, NB – default probability = 0.005.

Predicting 5-LO natural product inhibitors. A dataset of 24,500 commercially available natural products (an overlap between DNP and Zinc commercially available databases) was uploaded into KNIME analytics platform. Molecules were pre-processed and the descriptors calculated and normalised as in the training set. The implemented GBT, LR and NB models were used to predict labels for the test set, leading to 1,245 molecules predicted as positives by all three models and further 2,254 predicted by any two models (Full metrics in Table S1) for 5-LO, and 940 triple-positive predictions for FLAP dataset. All triple-positive and part of random (500 out of 2,254 double-positive 5-LO set molecules) were further investigated in PubChem database for their anti-inflammatory, anti-leukemia and anti-arthritic activities. Twelve and ten natural products from 5-LO and FLAP datasets with known anti-inflammatory, anti-

leukemic or anti-arthritic properties were chosen for further testing. Molecules described as 5-LO modulators in ChEMBL database were not chosen for testing.

Fragment analysis. 2,456 5-LO modulators were split into strong and weak modulator datasets as described above. Molecules in both sets were broken down into fragments with path lengths 4-8. Fragments containing interrupted aromatic systems were discarded. Fragment frequencies (number of molecules containing a particular fragment in a set versus a total number of molecules in a set) in both sets were calculated. Ratio Frequency(active)/Frequency(inactive) was used to calculate relative representation of each fragment in the active and inactive datasets.

pK_a predictions. pK_a values of resistomycin were predicted using MarvinView (ChemAxon/Infocom Marvin Extensions Feature 4.4.0.v211500) in KNIME Analytics Platform.

Expression and purification of human recombinant 5-LO. Human recombinant 5-LO was expressed in *E. coli* (BL21, DE3) transformed with pT3-5-LO plasmid at 30 °C overnight, as described before[6]. Cells were lysed using lysis buffer and homogenized by sonification (3 x 15 s, Branson Sonifier 250, Branson Ultrasonics Corporation). 5-LO was then purified from 40,000 x g supernatant (20 min, 4°C) using an ATP agarose column (Sigma-Aldrich), diluted with PBS (Dulbeco's formula, pH 7.4) buffer containing 1 mM EDTA, and immediately used for 5-LO activity assays.

Blood cell isolation. The process of isolation was performed as described by A. Boyum[7]. Briefly, peripheral blood was withdrawn from fasted healthy adult volunteers (University Hospital Jena, Germany) and centrifuged to obtain leukocyte concentrates. These were aliquoted and mixed with 2.5% dextran in PBS. After 45 min, leukocyte-rich supernatant was transferred to density centrifugation medium (Histopaque-1077; d = 1.077) and centrifuged (200 rpm, 10 min, room temperature, without brake, Heraeus Multifuge X3R Centrifuge, Thermo Fisher Scientific).

Peripheral blood mononuclear cells (PBMC) were concentrated on top of the density medium and separated from the other cells. Isolated PBMC were washed with PBS twice (1200 rpm, 5 min, 4 °C) and resuspended in 5 ml PBS.

For isolation of PMNL, contaminating erythrocytes of pelleted PMNL were removed by hypotonic lysis. Afterwards, PMNL were washed with PBS twice (1200 rpm, 5 min, 4 °C), resuspended in 5 ml PBS. A cell counting system (Vi-CellTMXR, Beckmann Coulter) was used to determine cell numbers and cell viability. For counting, the cell suspension was diluted (1:50) and trypan blue staining (0.4 % (v/v), sterile filtered) was used to determine cell viability.

Determination of 5-LO product formation using isolated enzyme. Isolated human 5-LO was diluted to optimal concentration (approx. 0.5 µg/ml) in 1 ml PBS containing 1 mM EDTA and treated with compounds or vehicle (0.1% (v/v) DMSO) for 10 min at 4 °C, and stimulated by addition of 2 mM $CaCl_2$ and 20 µM AA for 10 min at 37 °C. The reaction was stopped by adding 1 ml ice-cold methanol and samples were prepared for high performance liquid chromatography (HPLC) analysis.

Determination of 5-LO product formation in PMNL. Freshly isolated PMNL (5 x 10^6) were suspended in 1 ml PBS-glucose (Dulbeco's PBS with 1% w/v glucose) buffer and 1 mM $CaCl_2$ was added. Cells were treated with compounds or vehicle (0.1% (v/v) DMSO) for 10 min at 37 °C, and stimulated by addition of 2.5 µM A23187 (and 20 µM exogenous AA as stated in the text) for 10 min at 37 °C. The reaction was stopped by adding 1 ml ice-cold methanol and samples were prepared for HPLC analysis.

Solid phase extraction (SPE) and HPLC analysis. After stopping the 5-LO reactions (see above), 530 µl PBS-HCl (500 µl Dulbeco's PBS mixed with 30 µl of 1N HCl aqueous solution) and 200 ng internal standard PGB1 were added to the samples. The samples from PMNL incubations were centrifuged (870 x g, 10 min, 4 °C) before SPE was performed using reverse phase C18 SPE cartridges Clean-Up (United Chemical Technologies). Briefly, columns were conditioned with methanol and ddH_2O, samples were added, washed with ddH_2O and 25% (v/v) methanol, eluted with 300 µl methanol, mixed with 120 µl ddH_2O; 100 µl were then subjected to analysis. Major 5-LO products (LTB_4, all-trans isomers of LTB_4, and 5-H(p)ETE) were analyzed by RP-HPLC using a Nova-Pak C18 Radial-Pak Column (4 µm, 5 × 100 mm, Waters) under isocratic conditions (73% methanol/27% water/0.007% trifluoroacetic acid) at a flow rate of 1.2 mL/min and detected at 235 nm (for 5-H(p)ETE) or 280 nm (for LTB_4 and its all-trans isomers).

Lactate dehydrogenase (LDH) release assay. The LDH assay was performed using the CytoTox 96® Non-Radioactive Cytotoxicity Assay kit. Briefly, 2 x 10^5 PMNL or PBMC diluted in PBS-glucose buffer were seeded per well of a 96-well plate. Lysis control and 0.2% (v/v) triton X-100 were added to the cells and incubated for 45 min, compounds and vehicle (0.1% (v/v) DMSO) were added and incubated for 20 min at 37 °C. Stop solution was added, the plate was centrifuged (250 x g, 4 min, room temperature) and 50 µl of supernatant from each well was transferred. Afterwards, 50 µl of substrate mixture was added and incubated for 30 min at room temperature under exclusion of light. To finally stop the reaction, 50 µl of stop solution were added, the photometric measurement was done at 490 nm using a Multiscan Spectrum plate reader (Thermo Fisher Scientific). Cytotoxicity was calculated after background correction as:

$$lysis = \frac{A_{490}\ (compound)}{A_{490}\ (vehicle)} \times 100\%\ (5)$$

Hemolysis assay. Defibrinated OxoidTM sheep's blood (Thermo Fisher Scientific, Waltham, MA, USA) was diluted to a 5% (v/v) suspension in PBS. In a 96-well microtiter plate, 190 µl of blood suspension was added to 10 µl of compound in PBS containing 20 x stock. DMSO was diluted in PBS to a 20 x stock which after adding the blood gave a concentration of 0.1% (v/v, negative control). Addition of 1% (v/v) triton X-100 was used as a positive hemolysis control. Three replicates were performed for each compound concentration. The plate was then incubated at 37 °C for 2 or 24 h. Following the incubation, the plate was centrifuged (3,300 rpm, 5 min), 100 µl of supernatant were collected and transferred. To determine hemolysis, ultraviolet (UV) absorbance of free heme was measured immediately at 540 nm and

500 – 700 nm (spectrum) using a plate reader. Percentage of hemolysis was determined by:

$$hemolysis = \frac{A_{540}\ (compound) - A_{540}\ (PBS)}{A_{540}\ (1\%\ Triton\ X\text{-}100) - A_{540}\ (PBS)} \times 100\%\ (6)$$

MTT assay. Freshly isolated PBMC were diluted in RPMI supplemented with 10% fetal calf serum, 1% penicillin/streptomycin and 1 mM L-glutamine and 100 µl of the resulting cell suspension containing 2 x 10^5 cells were seeded per well in a 96-well plate. A 333-fold stock of compound or 0.3% (v/v) DMSO was added and incubated for 1 h or 24 h at 37 °C. As a positive control 16.7% (v/v) ethanol or 0.05% (v/v) triton-X were used, a negative control contained media only. Finally, 20 µl of MTT solution (5 mg/ml in PBS, sterile-filtered) was added per well and incubated for 3 h at 37 °C. Cells were lysed by addition of 100 µl SDS lysis buffer shaking at 175 rpm (neolab Multi shaker DOS-102, neoLab Migge) under exclusion of light overnight. The photometric measurement was performed at 400 – 600 nm (595 nm) using a Multiscan Spectrum plate reader (Thermo Fisher Scientific). Cell viability was calculated after background correction as:

$$cytotoxicity = \frac{A_{595}\ (compound)}{A_{595}\ (vehicle)} \times 100\%\ (7)$$

Cultivation and preparation of human pathogenic *E. coli*. Human pathogenic *E. coli* (O6:K2:H1 CFT073) was cultivated in 35 ml NB medium suspension in an Erlenmeyer flask shaking at 37 °C overnight. On the next day, 5 ml cell suspension were centrifuged (5000 x g, 5 min, 20 °C) and optical density OD600 was brought to 1.0 by diluting cells in PBS (pH 7.4) supplemented with 1 mM $CaCl_2$. OD_{600} was determined using a Ultrospec10-Cell density meter (Amersham Biosciences).

Differentiation and polarization of monocytes to M1 macrophages. Freshly isolated PBMC were cultivated in 15 ml PBS (Dulbeco's formula, supplemented with 100 mg/L $CaCl_2$ and $MgCl_2$ hexahydrate) in a 75 cm^3 cell culture flask at 37 °C for 1 h in the WTC Binder incubator (WTC Binder GmbH) in a humid and CO_2 enriched (5% v/v) atmosphere. After attachment of monocytes, cells in suspension were removed and 15 ml RPMI supplemented with 10% fetal calf serum, 1% penicillin/streptomycin and 1 mM L-glutamine medium were added per flask. For macrophage differentiation, 20 ng/ml GM-CSF was supplemented and after 6 days, cells were polarized with 20 ng/ml interferon-γ and 100 ng/ml LPS for 48 h at 37 °C and 5% (v/v) CO_2.

Stimulation of M1 macrophages and lipid mediator profiling by UPLC-MS/MS. M1 macrophages (1 x 10^6) were resuspended in 1 ml PBS (Dulbeco's formula, supplemented with 1 mM $CaCl_2$) and treated with test compounds or vehicle (0.1% (v/v) DMSO) for 20 min at 37 °C and 5% (v/v) CO_2. Afterwards, macrophages were stimulated by addition of *E. coli* (O6:K2:H1) at a ratio of 1:50 (M1:*E. coli* multiplicity of infection (MOI) of 50) for 90 min at 37 °C and 5% (v/v) CO_2. The reaction was stopped by transferring the supernatant to 2 ml ice-cold methanol and samples were prepared

for ultraperformance liquid chromatography–tandem mass spectrometry (UPLC-MS/MS) analysis.

For stimulation with Ca^{2+}-ionophore A23187, medium was removed, and macrophages (1 x 10^6) were treated by addition of compounds or vehicle (0.1% (v/v) DMSO) diluted in 1 ml PBS (Dulbeco's formula, supplemented with 1 mM $CaCl_2$) for 10 min at 37 °C and 5% (v/v) CO_2. Stimulation was performed by addition of 2.5 µM A23187 for 10 min at 37 °C and 5% (v/v) CO_2. The reaction was stopped by transferring the supernatant to 2 ml ice-cold methanol and samples were prepared for UPLC-MS/MS analysis.

To the samples, deuterated lipid mediators (d8-5S-HETE, d4-LTB_4, d5-LXA_4, d5-RvD2, d4-PGE_2; 200 nM each) and d8-AA (10 µM) were added as internal standards. Lipid mediators were extracted by solid phase extraction using Sep-Pak C18 6cc Vac Cartridges (500 mg; Waters), as previously described[8]. Briefly, samples were stored at -20 °C for at least 45 min to allow protein precipitation. After centrifugation (1200×g, 4 °C, 10 min), the supernatant was combined with acidified water (pH 3.5, 7 mL) and loaded onto pre-equilibrated solid phase cartridge columns. Washing steps with water and n-hexane (6 mL, each) followed. Lipid mediators were eluted with methyl formate (6 mL), brought to dryness using an evaporation system (TurboVap LV, Biotage, Uppsala, Sweden) and resuspended in methanol/water (50/50, v/v, 100 µL) for UPLC-MS/MS analysis.

For metabololipidomics analysis, lipid mediators were separated at 50°C on an Acquity UPLC BEH C18 column (130Å, 1.7 µm, 2.1 mm x 100 mm, Waters). The Acquity Ultraperformance LC system (Waters) was operated at a flow rate of 0.3 mL/min using a mobile phase consisting of methanol, water, and acetic acid (42:58:0.01, v/v/v), which was ramped to 86:14:0.01 (v/v/v) over 12.5 min followed by isocratic elution at 98:2:0.01 (v/v/v) for 3 min[8]. Eluted lipid mediators were detected by (scheduled) multiple reaction monitoring using a QTRAP 5500 mass spectrometer (Sciex, Framingham, MA), which was equipped with an electrospray ionization source that was operated in negative mode. Acquired mass spectra were processed using Analyst 1.6.2 (Sciex).

Determination of the intracellular Ca^{2+} concentration. The determination of the intracellular Ca^{2+} concentration was performed as previously described[9]. Briefly, 5 x 10^6 PMNL or 1 x 10^6 M1 macrophages were stained using 1 µM Fura-2-AM (30 min, 37 °C), washed with Krebs-HEPES buffer (centrifuged at 1000 rpm, 5 min, 4°C) and resuspended in 3 ml ice-cold Krebs-HEPES buffer plus BSA. 200 µl of cell suspension containing 5 x 10^5 PMNL or 2.5 x 10^5 M1 macrophages was seeded per well into a 96-well plate (black, clear bottom). Cells were incubated with 1 mM $CaCl_2$ for 10 min at 37 °C. To determine intracellular Ca^{2+} concentrations, a microplate fluorometer NOVOstar (BMG Labtech Optima) was used to measure the Ca^{2+}-dependent fluorescence of the intracellular dye over time at 37 °C. Fluorescence at 340 nm (Ca^{2+} chelating Fura-2-AM) and 380 nm (free Fura-2-AM) was measured every 1.18 s for 225 kinetic cycles (4.4 min total time). An automatic injecting system was used to perform the addition of the following solutions: 2 µl of 100-fold compound solution or

vehicle (1% (v/v) DMSO) were injected after 11.8 s, 20 µl of 10% (v/v) triton-X in Krebs-HEPES buffer plus BSA were injected after 218.3 s, and 16.6 mM of EDTA was added after 253.7 s. Each treatment was performed in duplicates. Intracellular Ca^{2+} was calculated from the ratio of fluorescence at 340/380 nm. Maximal Ca^{2+} release was calculated as:

$$[Ca^{2+}]_{max}\ (n\text{-}fold\ of\ CTRL) = \frac{[Ca^{2+}]_{\mathrm{max}(cmpd)} - [Ca^{2+}]_{\mathrm{basal}(cmpd)}}{[Ca^{2+}]_{\mathrm{max}(vehicle)} - [Ca^{2+}]_{\mathrm{basal}(vehicle)}} \times 100\% \ (8)$$

Determination of intracellular ROS formation. The detection of ROS was conducted using the peroxide-sensitive fluorescence dye DCFH-DA. PMNL were diluted to 5 x 10^6 cells/ml in PBS-glucose buffer and 100 µl per well were seeded into a 96-well plate (black, clear bottom). Compound, vehicle (2% (v/v) DMSO) or DPI (positive control inhibitor of ROS formation) and 100 µl of ROS measuring solution (2 µg/ml DCFH-DA, 2 mM $CaCl_2$, in PBS-glucose buffer) were added and incubated for 10 min at 37 °C under exclusion of light. Afterwards, either PBS-glucose buffer or 1 µM PMA was added and ROS formation was measured immediately (t0) at 37 °C using microplate fluorometer NOVOstar (BMG Labtech Optima). Excitation occurred at 485 nm, where emission was measured at 520 nm every 6 s for 150 kinetic cycles (15 min total time). The detection level was reached after 600 s and thus, evaluation was performed after 480 s (t1) when the curve was still in the linear range. ROS formation was calculated from fluorescence change (t1 – t0) as percentage of vehicle control, values of cells treated with vehicle and stimulated with PMA were set as 100%. Calculation was performed as:

$$ROS\ formation\ (\%\ of\ CTRL) = \frac{F_{520\ (t1)\ cmpd} - F_{520\ (t0)\ cmpd}}{F_{520\ (t1)\ vehicle} - F_{520\ (t0)\ vehicle}} \times 100\% \ (9)$$

Statistical Analysis. Data are presented as mean ± S.D. of n observations, where n represents the number of independent experiments performed at different time points. General statistical analyses were carried out using a two-sided Student's t-test at a confidence interval of 95%.

References

(1) Fillbrunn, A.; Dietz, C.; Pfeuffer, J.; Rahn, R.; Landrum, G. A.; Berthold, M. R. KNIME for reproducible cross-domain analysis of life science data. *J. Biotechnol.* **2017**, *261*, 149–156.

(2) Friedman, J. H. Greedy function approximation: A gradient boosting machine. *Ann. Stat.* **2001**, *29* (5), 1189-1232.

(3) Dreiseitl, S.; Ohno-Machado, L. Logistic regression and artificial neural network classification models: a methodology review. *J. Biomed. Inform.* **2002**, *35* (5-6), 352–359.

(4) Wickramasinghe, I.; Kalutarage, H. Naive Bayes: applications, variations and vulnerabilities: a review of literature with code snippets for implementation. *Soft Computing* **2021**, *25* (3), 2277–2293.

(5) Bender, A.; Schneider, N.; Segler, M.; Walters, W. P.; Engkvist, O.; Rodrigues, T. Evaluation guidelines for machine learning tools in the chemical sciences. *Nat. Rev. Chem.* **2022**, *6* (6), 428–442.

(6) Fischer, L.; Szellas, D.; Radmark, O.; Steinhilber, D.; Werz, O. Phosphorylation- and stimulus-dependent inhibition of cellular 5-lipoxygenase activity by nonredox-type inhibitors. *FASEB J.* **2003**, *17* (3), 949–951.

(7) Boyum, A. Isolation of mononuclear cells and granulocytes from human blood. Isolation of monuclear cells by one centrifugation, and of granulocytes by combining centrifugation and sedimentation at 1 g. *Scand. J. Clin. Lab. Invest. Suppl.* **1968**, *97*, 77.

(8) Werner, M.; Jordan, P. M.; Romp, E.; Czapka, A.; Rao, Z. G.; Kretzer, C.; Koeberle, A.; Garscha, U.; Pace, S.; Claesson, H. E.; et al. Targeting biosynthetic networks of the proinflammatory and proresolving lipid metabolome. *FASEB Journal* **2019**, *33* (5), 6140–6153.

(9) Grynkiewicz, G.; Poenie, M.; Tsien, R. Y. A new generation of Ca2+ indicators with greatly improved fluorescence properties. *J. Biol. Chem.* **1985**, *260* (6), 3440–3450.
